# Real-Time Assessment of Rodent Engagement Using ArUco Markers: A Scalable and Accessible Approach for Scoring Behavior in a Nose-Poking Go/No-Go Task

**DOI:** 10.1523/ENEURO.0500-23.2024

**Published:** 2024-03-01

**Authors:** Thomas J. Smith, Trevor R. Smith, Fareeha Faruk, Mihai Bendea, Shreya Tirumala Kumara, Jeffrey R. Capadona, Ana G. Hernandez-Reynoso, Joseph J. Pancrazio

**Affiliations:** ^1^School of Behavioral and Brain Sciences, The University of Texas at Dallas, Richardson, Texas 75080; ^2^Department of Mechanical and Aerospace Engineering, West Virginia University, Morgantown, West Virginia 26506; ^3^Department of Bioengineering, The University of Texas at Dallas, Richardson, Texas 75080; ^4^Department of Biomedical Engineering, Case Western Reserve University, Cleveland, Ohio 44106; ^5^Advanced Platform Technology Center, Louis Stokes Cleveland Veterans Affairs Medical Center, Cleveland, Ohio 44106

**Keywords:** ArUco markers, automated scoring, computer vision, engagement analysis, go/no-go, real-time tracking

## Abstract

In the field of behavioral neuroscience, the classification and scoring of animal behavior play pivotal roles in the quantification and interpretation of complex behaviors displayed by animals. Traditional methods have relied on video examination by investigators, which is labor-intensive and susceptible to bias. To address these challenges, research efforts have focused on computational methods and image-processing algorithms for automated behavioral classification. Two primary approaches have emerged: marker- and markerless-based tracking systems. In this study, we showcase the utility of “Augmented Reality University of Cordoba” (ArUco) markers as a marker-based tracking approach for assessing rat engagement during a nose-poking go/no-go behavioral task. In addition, we introduce a two-state engagement model based on ArUco marker tracking data that can be analyzed with a rectangular kernel convolution to identify critical transition points between states of engagement and distraction. In this study, we hypothesized that ArUco markers could be utilized to accurately estimate animal engagement in a nose-poking go/no-go behavioral task, enabling the computation of optimal task durations for behavioral testing. Here, we present the performance of our ArUco tracking program, demonstrating a classification accuracy of 98% that was validated against the manual curation of video data. Furthermore, our convolution analysis revealed that, on average, our animals became disengaged with the behavioral task at ∼75 min, providing a quantitative basis for limiting experimental session durations. Overall, our approach offers a scalable, efficient, and accessible solution for automated scoring of rodent engagement during behavioral data collection.

## Significance Statement

This paper presents an accessible and effective solution for automating the scoring of rodent engagement during a go/no-go nose-poking behavioral task. Here, we showcase the effectiveness of implementing a marker-based tracking approach by mounting ArUco markers to the animal's head and using webcams with open-source tracking software to reveal transition points between states of engagement and distraction throughout a behavioral session. This approach offers a significant advancement for simplifying the scoring process with marker-based tracking while also showing promise in broad applicability toward quantifying optimal task durations for various behavioral paradigms. By making this software freely available, we aim to facilitate knowledge exchange, encourage the scientific community's engagement, and expand its application across diverse research endeavors.

## Introduction

In behavioral neuroscience, the classification and assessment of animal behavior are crucial for comprehending and quantifying the complex routines displayed by animals. Traditional methods for behavioral analysis have often relied on labor-intensive video examination, which can be both time-consuming and susceptible to bias (Marsh and Hanlon, 2007; von Ziegler et al., 2021). In response to these challenges, recent work has sought innovative approaches that leverage computational methods, specifically those employing image-processing algorithms (Walter and Couzin, 2021; Isik and Unal, 2023). To automate the process of behavioral analysis, two predominant approaches have emerged: marker- and markerless-based tracking systems (Mathis et al., 2018; Bala et al., 2020; Haalck et al., 2020; Moro et al., 2022; Pereira et al., 2022). Each method, however, possesses its own advantages and disadvantages when capturing, quantifying, and classifying animal movement behavior.

Recent trends in behavioral tracking have favored markerless systems, particularly those based on machine-learning algorithms for pose estimation (Nath et al., 2019; Liang et al., 2021). These systems rely on the inherent features of the animals themselves to serve as reference points, eliminating the need for attached physical markers. Altogether, they offer an advantage in achieving strong consistency while simultaneously tracking multiple points on an animal's body for analysis of complex behaviors without hindering movement (Mathis et al., 2018; Nath et al., 2019; Pereira et al., 2022). However, these advanced systems introduce several challenges, including the need for high-end equipment to meet computational demands, as well as the acquisition of extensive training data to construct a robust model (Mathis et al., 2018; Pereira et al., 2022). For example, the creators of the leading markerless tracking system, DeepLabCut, recommend using an NVIDIA GPU with a minimum of 8 GB of VRAM for training models (Mathis et al., 2018). In accordance with their GitHub repository documentation, failure to utilize such GPUs or relying on CPU alone can result in significantly slower execution with a performance difference of approximately 10×. Moreover, markerless systems depend on the presence of numerous reference points for computing accurate orientation data. As a result, increasing the number of tracked subjects often amplifies computational demands as it needs to differentiate which points belong to each subject (Walter and Couzin, 2021; Pereira et al., 2022). For each of these reasons, markerless approaches are usually employed as a post-session analysis tool to alleviate the burden of real-time processing demands.

In contrast, marker-based tracking systems offer a simpler yet potentially effective solution. A marker-based tracking algorithm relies on detecting and tracking physical markers that can be placed/mounted on the subject (Menolotto et al., 2020; Moro et al., 2022; Vagvolgyi et al., 2022). Unlike markerless pose estimation, a single marker design can hold sufficient information for estimating the position and orientation of a subject within an image or video (Garrido-Jurado et al., 2014; Sampathkrishna, 2022). One notable example is the ArUco marker, named for “Augmented Reality University of Cordoba,” developed in 2014 by the University of Cordoba, Spain (Garrido-Jurado et al., 2014). ArUco markers are physical markers that consist of black-and-white square grid patterns, serving as fiducial target markers – objects that are easily detected and tracked by cameras. Since ArUco markers do not rely on machine learning or the need for prior training data, this approach inherently requires minimal computational resources for real-time analyses (Garrido-Jurado et al., 2014). For example, a low-end CPU-driven system with an Intel Core 2 Quad 2.40 GHz processor and 2,048 MB of RAM is readily capable of detecting an ArUco maker under the open-source Ubuntu operating system (Garrido-Jurado et al., 2014). This ensures that ArUco markers can enable real-time tracking at 60 frames per second (fps) while making it feasible and accessible to track multiple markers simultaneously with over 100+ uniquely identifiable patterns in the ArUco library.

In the present work, we investigated the utility of ArUco markers to automatically score animal engagement (e.g., “engaged” or “distracted”) during a rat nose-poking go/no-go task where the animals received electrical stimulation via an intracortical microelectrode array. We show that ArUco markers can reliably estimate animal engagement in the go/no-go task with high accuracy and precision. Furthermore, this approach demonstrated an adjustable method for estimating the time point at which an animal begins to disengage from their behavioral task, providing a quantitative basis for limiting experimental session durations. Altogether, we provide an accessible, scalable, and precise procedure for real-time automated scoring of rodent engagement during behavioral data collection. To enhance transparency, we have made the code/software, 3D models, and data essential for replicating our results accessible as Extended Data. These resources can also be obtained online for free at https://github.com/tomcatsmith19/ArucoDetection.

## Materials and Methods

### Animal use

All animal handling, housing, and procedures were approved by The University of Texas at Dallas IACUC (protocol #21-15) and in accordance with ARRIVE guidelines (Percie du Sert et al., 2020). We used three (*N* = 3) male Sprague-Dawley rats (Charles River Laboratories Inc.) individually housed in standard home cages under a reverse 12 h day/night cycle that began at 6:00am. Behavioral experiments were performed during the night period (Zeitgeber Time 12–24). To ensure consistent engagement during behavioral sessions, we slightly modified an established mild food deprivation regime with ad libitum access to water (Schindler et al., 1993). Each rat was maintained at a target weight of 90% of their free-feeding levels which was adjusted weekly according to weight. Friday night through Monday morning, animals had access to food ad libitum. During behavioral testing, dustless reward pellets (F0021, Bio-Serv) were used as positive reinforcement, ensuring nutritional balance. Between sessions, we provided supplemental food pellets (5LL2 - Prolab RMH 1800, LabDiet) that were adjusted based on reward pellet consumption.

### Surgical procedure

All rats underwent a surgical procedure for a microelectrode array implantation that followed established protocols. Briefly, rats were anesthetized with isoflurane (1.8–2.5%) mixed with medical-grade oxygen (500 ml/min, SomnoSuite for Mice & Rats, Kent Scientific Corporation). Vital signs were monitored throughout the procedure, and body temperature was maintained using a far-infrared warming pad (PhysioSuite for Mice & Rats, Kent Scientific Corporation). The surgical area was prepared using three alternating applications of povidone-iodine (PVP Iodine Prep Pads, Dynarex) and 70% isopropyl alcohol wipes (Isopropyl Alcohol Swabs, Becton Dickinson). Next, a subcutaneous injection of 0.5% bupivacaine hydrochloride (Marcaine, Hospira) was administered followed by a 2.5–3 cm incision through the scalp, muscles, and connective tissue. Three burr holes were drilled into the skull using a motorized drill (H.MH-170 Rotary Handpiece, Foredom Electric Company) to anchor stainless steel bone screws (1.59 mm O.D., 3.2 mm long, Stoelting Co.). A 2 mm × 3 mm craniotomy was made targeting the left somatosensory cortex (AP, −0.5 mm; ML, 4 mm), followed by a durotomy. Microelectrode arrays were implanted to a depth of 1.6 mm for one animal and 2.0 mm for the remaining two animals using a precision inserter (NeuralGlider, Actuated Medical, Inc.) at a speed of 0.1 mm/s with actuation on. When possible, the disruption of superficial blood vessels was avoided (Kozai et al., 2010). The implant site was sealed with silicone elastomer adhesive (Kwik-Sil, World Precision Instruments), followed by a dental cement head cap. The incision was closed with surgical staples (Autoclip Wound Clips, 9 mm, Becton Dickinson) and tissue adhesive (GLUture, World Precision Instruments). After surgery, we injected each animal with 0.05 ml/kg intramuscular cefazolin (SKU: 054846, Covetrus) as antibiotic prophylaxis, 0.5 ml/kg of extended-release buprenorphine (Ethiqa XR, Fidelis Animal Health) as an analgesic, and topical triple-antibiotic ointment around the closure site. Sulfamethoxazole and trimethoprim oral suspension liquid (200 mg/40 mg/5 ml, Aurobindo Pharma) was provided in their drinking water (1 ml/100 ml drinking water) for 7 d postsurgery.

### Marker mounting hardware

**Figure 1.**
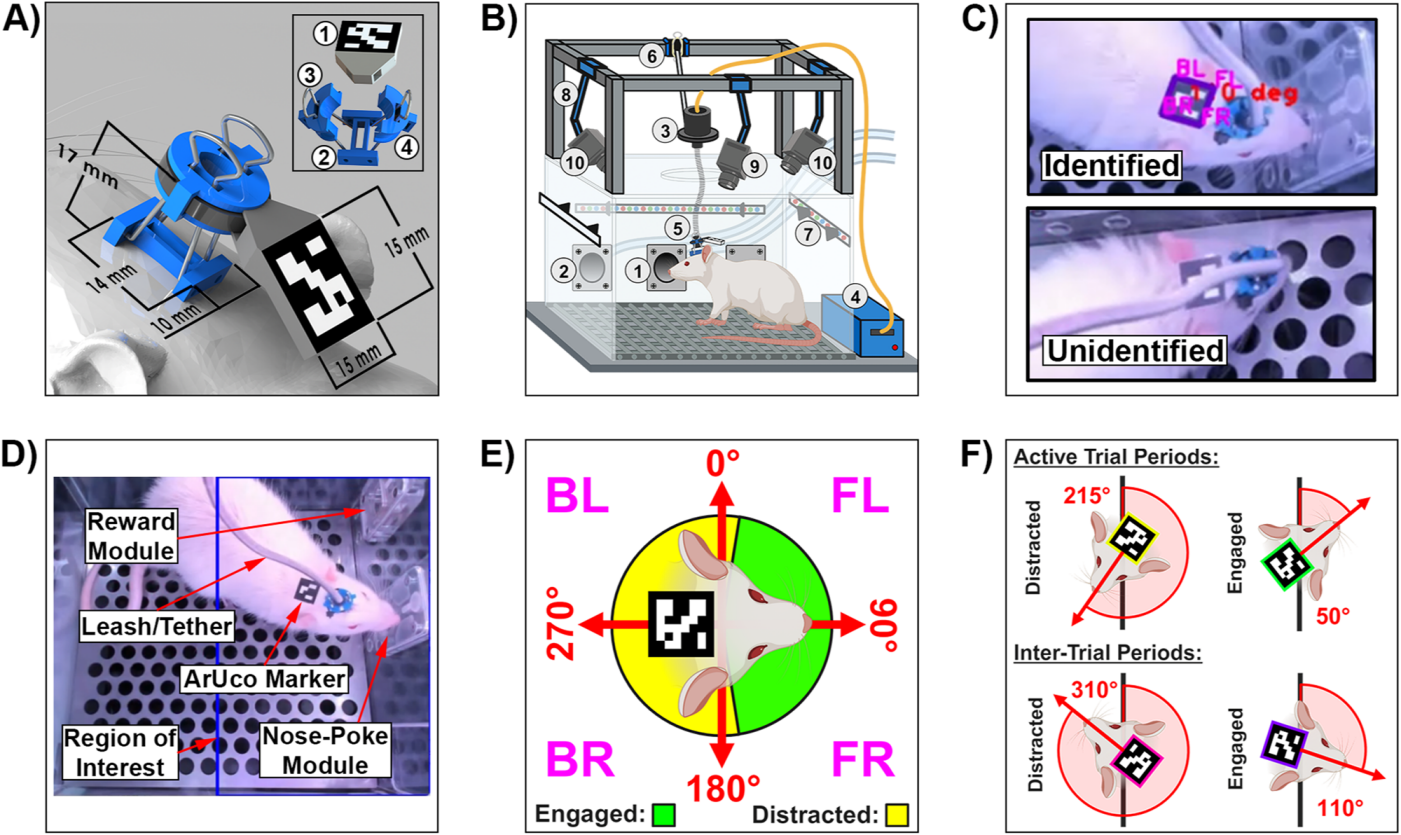
ArUco tracking hardware and classification parameters. ***A***, Dimensions of the ArUco marker mounting assembly as positioned on a 3D-rendered rat. ***B***, Behavioral chamber components. ***C***, Instance of an identified ArUco marker through the custom Python ArUco marker tracking program, as well as an example of occlusion where the marker could not be identified. ***D***, Overhead display of a rat inside of the behavioral chamber with the region of interest for engagement outlined in blue. ***E***, ArUco tracking parameters, based on the yaw angle, for engagement and distraction classification while inside the region of interest. ***F***, Examples of distraction and engagement classifications, during active and inter-trial periods.

We designed a 3D-printed custom mounting assembly to securely mount and unmount the ArUco marker to the rat's headcap while tethered (Fig. 1*A*). Details about the 3D-printed components, which were created using polylactic acid (PLA) filament, and the other assembly materials, can be found in Table 1. The 15 mm  × 15 mm ArUco marker was printed on standard white paper and glued to the marker mount (Fig. 1*A*.1). The i-bracket was a low-profile 3D-printed object that was permanently glued to an animal's headcap (Fig. 1*A*.2), featuring strategically positioned holes on each side-protrusion for quick insertion/removal of the 15 mm binder clip wires (Fig. 1*A*.3). Additionally, the rectangular center hole and height of the i-bracket were specifically designed to surround our microelectrode array's connector port (A79016-001, Omnetics Connector Corporation) for accessible attachment of the tether to the animal. Next, the c-clamps were 3D-printed pieces that wrapped around the binder clip wires to grip the tether firmly and avoid detachment during behavior (Fig. 1*A*.4). The 4" cable ties served a dual purpose when threaded through the ArUco marker mount: (1) securing the ArUco marker mount to the rest of the mounting assembly and (2) constricting the c-clamps around the tether. This meticulous arrangement ensured that the ArUco marker remained positioned in the midline of the rat's neck, offering a typically unobstructed line of sight to the overhead webcams. In total, the marker's position served as an approximation for tracking head movement that remained reliably affixed throughout the study without impeding animal performance, thus contributing to the accuracy of the research. All 3D-printed files are provided on our GitHub repository for the study at https://github.com/tomcatsmith19/ArucoDetection.

**Table 1.** ArUco marker mounting assembly components list

Component name	PLA filament usage (g)	Estimated price (2023)
15 mm × 15 mm ArUco marker	N/A	<$1.00
ArUco marker mount	0.8	$0.02
i-bracket	0.3	$0.01
C-clamp (1×)	0.3	$0.01
15 mm binder clip wire (1×)	N/A	<$1.00
4' cable tie	N/A	<$1.00
Total		<$3.00

Prices for each of the 3D-printed pieces were estimated based on the approximate $20 cost of a standard 1.75 mm PLA 3D Printer Filament 1 kg (2.2 lbs) Spool in 2023. Filament usage measurements in grams were calculated using a 3D-printing slicer software (Ultimaker Cura, V4.13.0) with the layer height set to 0.2 mm and cubic pattern infill density of 20%.

**Table 2.** Computational resource usage benchmarks

Configuration and task	Approximate CPU usage	Approximate memory usage
Idle computer on desktop	2% (1.0 GHz)	12% (8 GB)
Behavioral program with single-camera configuration	28% increase from Idle (2.2 GHz)	8% increase from Idle (5.3 GB)
Behavioral program with double-camera configuration	28% Increase from Idle (2.2 GHz)	8% Increase from Idle (5.3 GB)

This table presents the average computational burden imposed by the ArUco marker tracking program during a live behavioral session for both single- and double-camera configurations. Values were monitored using the performance tab under the task manager application in Windows 11.

### Nose-poking go/no-go behavioral paradigm and operant conditioning chamber

The behavioral methods and equipment configurations utilized in this study were based on an established and validated paradigm (Smith et al., 2023). Briefly, animals were trained to participate in an established nose-poke, go/no-go behavioral paradigm to evaluate stimulation-evoked perception thresholds in rats. In the go/no-go paradigm, rats were trained to distinguish between “go” signals (varying magnitudes of ICMS ranging from 0 to 4 nC/ph equivalent to 0–20 µA) and “no-go” signals (e.g., absence of stimulation). If the rats perceived the stimulus, they were trained to nose-poke into a designated hole on a chamber wall to receive a sugar pellet reward (F0021, Bio-Serv). If a stimulus was not presented or perceived, then they were trained not to poke. At all times, one of three LED light colors filled the behavioral chamber to indicate trial status: (1) green lights indicated when an active trial was taking place, (2) red lights displayed when a punishment was being administered, and (3) white lights represented an inter-trial period where nose-pokes were negated/ignored. All active trials lasted a duration of 6 s, followed by an inter-trial period of 3 s. Behavioral sessions were condoned 4 d a week with a condensed session time of 32 min per day compared to the original duration of 60 min.

Figure 1*B* depicts the behavioral chamber utilized in this study. The go/no-go behavioral paradigm was conducted within a commercially available operant conditioning chamber (OmniTrak, Vulintus, Inc.). This chamber featured two side wall holes, one equipped with an infrared break-beam sensor (nose-poke module; Fig. 1*B*.1), and the other connected to a precision pellet dispenser (reward module; Fig 1*B*.2). A rotating commutator (AC6023-18, Moog, Inc.; Fig. 1*B*.3) was installed to allow animals freedom of movement while connected to an external stimulator (PlexStim, Plexon Inc.; Fig. 1*B*.4) used for intracortical microstimulation delivery. To safeguard against wire damage via biting, a custom leash/tether cord (Fig. 1*B*.5) was micro-constructed using an Omnetics adapter (A79021-001, Omnetics Connector Corporation) and stainless steel spring cable shielding (#6Y000123101F, Protech International Inc.). Furthermore, a custom-built cable tensioner (Fig. 1*B*.6) was utilized to keep the tether taut, minimizing the rats’ ability to pull and bite on the leash. Next, chamber illumination was managed by an RGB LED strip fastened around the chamber walls (Fig. 1*B*.7). Three webcams were mounted to a 3D-printed articulated webcam mount (Articulating Arms with holder for T & V slot and knobs, danid87, Thingiverse; Fig. 1*B*.8) that was then affixed to the chamber. One webcam was used to record general animal behavior from the back of the chamber at 30 fps (JVCU100, j5create; Fig. 1*B*.9), while the remaining two were mounted to the right (Camera #1) and left (Camera #2) sides of the chamber facing inward and used to track ArUco markers at 60 fps 1,080p resolution (960-001105, Logitech; Fig. 1*B*.10). All components of the chamber were linked and governed by an ATMEGA2560 microcontroller board hub (OmniTrak Controller V3.0, Vulintus Inc.) and Dell Precision 5860 Workstation computer (Dell) that was interfaced using custom MATLAB (R2023b, MathWorks) and Python (V3.10.11, Python Software Foundation) software. A complete configuration list for the Dell workstation can be found on our GitHub repository at https://github.com/tomcatsmith19/ArucoDetection. Lastly, a custom MATLAB GUI application was developed to control task parameters, monitor performance, and record session data in real-time, allowing for trial annotation and offline validation.

### Marker detection and classification of engagement

A bi-directional network communication data stream between MATLAB and Python language environments was used to employ a unified behavioral and ArUco marker tracking program, which was achieved through the use of the open-source socket library (V3.12). This configuration allowed each environment to act as both client and server on a single workstation computer. Internally, execution of the MATLAB behavioral code would trigger a socket stream write command at the beginning and end of each trial where a data value would be updated and readily accessible within the Python environment through a socket stream read command. Depending on the value, the ArUco classification functions were either initiated or terminated. Upon detecting a termination signal at the end of each trial, a subsequent Python command would reverse the signaling process, sending a value back to MATLAB as a representation of the classification result for the trial. This synchronization structure ensured seamless data collection with minimal adaptations added to the established go/no-go software operating within the MATLAB environment.

For ArUco marker detection, our Python programs utilized the Open Source Computer Vision Library (OpenCV) software (V4.7.0), which offers a comprehensive toolkit for tasks related to computer vision, image processing, and machine learning. Notably, OpenCV comes equipped with an integrated ArUco marker detection feature (Hu et al., 2019; Oš̌ádal et al., 2020), which was implemented for identifying the location and orientation of the ArUco markers within captured video frames. This detection process relies on analyzing one of the many unique patterns of ArUco markers, by combining pattern recognition, corner detection, and pose estimation techniques (Garrido-Jurado et al., 2014; Sarmadi et al., 2019; Čepon et al., 2023). However, detection relies on markers being displayed within the camera's direct line of sight (Fig. 1*C*). Should the marker be visually obstructed by factors such as occlusion, unfavorable lighting conditions, or motion blur caused by rapid animal movement, then it may become unidentifiable, potentially resulting in incorrect trial classifications of distraction (Garrido-Jurado et al., 2014; Hu et al., 2019; Sampathkrishna, 2022). To counter instances of occlusion and motion blur, we combined the perspectives of both 60 fps webcams mounted on the chamber for better marker detection. Furthermore, we use the chamber's RGB lights to keep the marker illuminated above the minimum brightness requirements of three lux (Hu et al., 2019).

The ArUco tracking program was used to automatically classify animal engagement in the go/no-go paradigm. This assessment was based on both the marker's location and orientation within a specified region of interest inside the behavioral chamber. In this work, only one region of interest was utilized. However, we have incorporated the ability to add multiple regions of interest and independently modify existing regions for each camera within the Python program called “AnimalDetect.py.” Here, our region of interest extended from the midline of the behavioral chamber's floor to the wall containing the nose-poke and reward modules (Fig. 1*D*; right-hand side of chamber). If the marker was found within this region of interest and was oriented in such a direction that the marker's edge closest to the animal's nose was facing the chamber's module wall (within a yaw angle range of 5°–175°; 90° indicating orthogonality between the marker edge and wall), then we classified the animal as “engaged” (Fig. 1*E*). Any orientation of the marker found outside of this range, regardless of location, resulted in a behavioral classification of “distracted.” A broader yaw angle range for engagement was chosen to give the animal more leeway in terms of orientation. However, situations where the animals fell on the border of these parameters might have also led to incorrect classifications depending on how the expert evaluators interpreted the animal's behavior. During active trial periods, the program outlined the ArUco marker in green when the animal appeared to be engaged with the task. When distractions were detected, the outline shifted to yellow, providing immediate visual feedback to the investigator. During the inter-trial periods, the marker's outline transitioned to pink when distractions were detected and purple when the animal was engaged. Examples of each situation can be found in Figure 1*F*. Moreover, if the animal met the parameter requirements for engagement at any time during an active trial, regardless of instances of distraction, then the trial was scored as engaged. However, since the tracking program was not yet capable of distinguishing between the distracting behaviors of locomotion, grooming, rearing, itching, etc., incorrect classifications of engagement could be seen if the marker was detected in a position of engagement during that period.

To verify the model's accuracy, we validated the classification results of the ArUco tracking program against the human expert scoring of animal behavior conducted by two independent investigators. The two investigators manually scored each behavioral trial independently, via post-session video analysis, as either engaged or distracted; any differences between them were re-analyzed, discussed, and agreed upon by both individuals.

### Data analysis and statistics

All computer-generated data analysis was conducted through custom MATLAB (R2023b) and Python (V3.10.11) programs, alongside GraphPad Prism (v10.0.2, GraphPad Software) as the primary statistical software. First, we compiled the classification data generated through the ArUco tracking program and validated it against the human-generated control data, focusing on instances where the animal did not nose-poke, which required judgments to be made under engagement uncertainty. This comparison involved the creation of a confusion matrix which included the following elements: true positives (TP), true negatives (TN), false positives (FP), and false negatives (FN), where positives indicated engaged trials and negatives represented distracted trials. From that data, we used a custom MATLAB algorithm to calculate the accuracy (Eq. 1), precision (Eq. 2), sensitivity (Eq. 3), specificity (Eq. 4), and F1-score (Eq. 5) metrics of the matrix, outlining the overall accuracy of the model in relation to manual analysis (Baratloo et al., 2015).

(1) Accuracy=(TP+TN)/(TP+TN+FP+FN)*100,

(2) Precision=(TP)/(TP+FP)*100,

(3) Sensitivity=(TP)/(TP+FN)*100,

(4) Specificity=(TN)/(TN+FP)*100,

(5) $$F1=2\;\left(\frac{\mathrm{Precision}\;*\;\mathrm{Sensitivity}\;}{\mathrm{Precision}+\mathrm{Sensitivity}}\right)\;*\;100.$$

To compute consistency in ArUco marker tracking throughout a behavioral session, we implemented a custom Python program that calculated the total number of video frames where the marker was identified by Camera #1 exclusively, Camera #2 exclusively, both cameras, or neither. Additionally, we compared the average computing resource benchmarks of the workstation's CPU and memory usage between both camera setups by monitoring the performance tab in Windows Task Manager while running the behavioral paradigm. Since our program is not GPU-dependent, its performance metrics were negated. Next, we used a digital luxmeter (HRD-PN-79081807, Leaton) to measure the minimum brightness values, in lux, produced inside the chamber throughout the varying light conditions of the behavioral paradigm. Lastly, to determine the velocity range of which the ArUco marker could still be identified within our setup, we used Equation 6 in reference to the experiments conducted in (Hu et al., 2019). Here, velocity in cm/s was derived as the product of the blur kernel size in pixels, frame rate of the camera (fps), and the camera's field of view width (cm) divided by the camera's resolution width (pixels).

(6) $$\mathrm{velocity}=\mathrm{blur}\;\mathrm{kernel}\;\mathrm{size}\;*\;\mathrm{frames}\;\mathrm{per}\;\mathrm{second}\;*\;\frac{\mathrm{field}\;\mathrm{of}\;\mathrm{view}\;\mathrm{width}}{\mathrm{resolution}\;\mathrm{width}}.$$

For behavioral optimization, a back-heavy rectangular kernel convolution was implemented to analyze the dynamics of behavioral engagement over time in a nose-poking go/no-go paradigm. In our convolution, a 5 min kernel window with a 1 min stepping pattern was used in MATLAB to estimate transition points between states of engagement and distraction. This analysis was applied to two different permutations of the behavioral task: (1) a typical 32 min session and (2) an extended 2-h-long version of the same behavioral session. In this study, the animals were considered to be fully disengaged from the task once their probability of engagement (Eq. 7) fell below a threshold of 50%.

(7) probabilityofengagement=(#ofengagedtrials/#oftotaltrials)*100.

For statistical analysis, the Matthews correlation coefficient (MCC) – a statistic used in machine learning and statistical analysis to measure the quality of binary classification models, particularly when dealing with imbalanced datasets – was calculated in MATLAB (Eq. 8) to comprehensively evaluate the overall performance of the ArUco tracking program by comparing its correlation to the human-generated control data (Chicco, 2017; Chicco and Jurman, 2020).

(8) $$\mathrm{MCC}=(\mathrm{TP}\;*\;\mathrm{TN}\text{-}\mathrm{FP}\;*\;\mathrm{FN})/\sqrt{\left(\mathrm{TP}+\mathrm{FP})(\mathrm{TP}+\mathrm{FN})(\mathrm{TN}+\mathrm{FP})(\mathrm{TN}+\mathrm{FN}\right)}\;.$$

This assessment is particularly relevant for our data, considering that our animals are actively participating in an established behavioral paradigm designed to maintain rats’ nose-poking, therefore skewing our data distribution toward trials of engagement. In addition, GraphPad Prism was used to calculate a one-tailed paired t-test for comparing the number of video frames where an ArUco marker was identified between single- and double-camera setups. Lastly, tests of normality in the data were analyzed using the Shapiro–Wilk test and confirmed results by examination of their respective Q-Q plots. All statistical results are reported as the mean ± SEM. We defined statistical significance as *p* < 0.05.

### Code/software accessibility

The code/software described in the paper is freely available online at https://github.com/tomcatsmith19/ArucoDetection. The code is also available as Extended Data.

10.1523/ENEURO.0500-23.2024.d1-1 Repository Files **Extended Data 1. GitHub Repository Code and Additional Files.** This file contains all of the code/software, 3D models, and files that will be provided in the GitHub repository for running the experiments and analyzing the data. Download Repository Files, ZIP file.

### Data accessibility

The raw data supporting the conclusions of this article will be made available by the authors, without undue reservation.

## Results

### Classification performance

**Figure 2.**
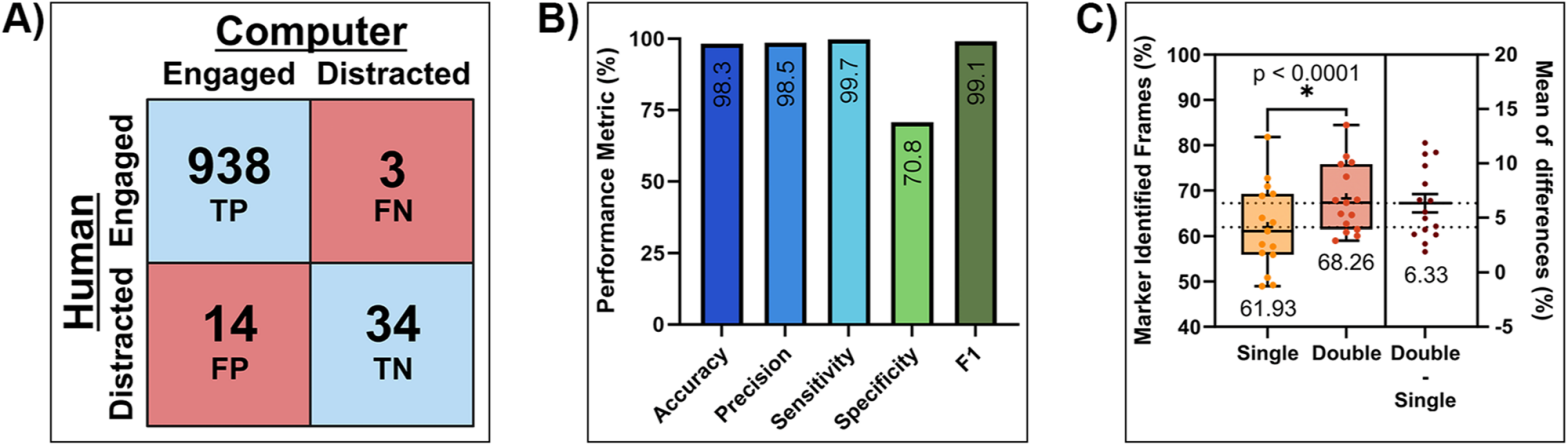
ArUco tracking performance results. ***A***, A confusion matrix showing individual trial scoring for human-generated analysis (rows) and ArUco tracking program classifications (columns). Values depicted represent data gathered across 15 behavioral sessions with five sessions per animal. ***B***, ArUco tracking program performance metrics calculated from confusion matrix data. ***C***, Results showcasing average differences in the number of total frames where the ArUco marker was identified during a behavioral session when a single- and double-camera configuration was used. Box plots (left) show error bars for min/max values, a center horizontal line for median values, and a plus sign for average values. The horizontal line and error bars in the right plot signify average ± SEM. Significance was defined as *p* < 0.05.

In this study, we scored instances of engagement for three distinct animals across five 32-min go/no-go behavioral sessions each, accumulating a total of 989 analyzed trials. During the manual scoring process, 941 trials (95.15%) were classified as “engaged,” while 48 trials (4.85%) were classified as “distracted.” Within this dataset, only four trials were disputed and then reconciled between the two expert evaluators. Overall, this manually generated analysis displayed a skew toward engagement, indicating that, during 32 min sessions, the animals were primarily focused on their behavioral tasks. In comparison, the ArUco tracking program, when applied to the same subset of behavioral data, scored 952 trials (96.3%) as “engaged” and 37 trials (3.7%) as “distracted,” respectively. Within the corresponding confusion matrix for this data (Fig. 2*A*), we calculated an accuracy of 98.3%, precision of 98.5%, sensitivity of 99.7%, specificity of 70.8%, and F1-score of 99.1% indicating high classification metrics for this specific behavioral paradigm in everything except specificity (Fig. 2*B*). This indicates that the current methodology could accurately identify as “engaged” those instances when the animal was engaged in the task but could only moderately identify as “distracted” those instances when the animal was in fact distracted. However, we found that the decrease in specificity could be attributed to instances of false positives, where the animal was grooming within the region of interest while the marker was scored as engaged; this accounted for 100% of the false positive occurrences. Despite these issues, the value obtained from the MCC test (0.80), indicated a strong positive correlation between the ArUco model's classifications and the human-generated control data. Overall, this suggests that our model still demonstrated strong predictive accuracy and reliability when identifying animal engagement.

### ArUco detection performance

Throughout the behavioral paradigm, the minimum brightness values measured under three potential light conditions were approximately 27 lux for red, 52 lux for green, and 62 lux for white light, all deemed sufficient for ArUco marker detection (Hu et al., 2019). In addition to these measurements, we conducted comparisons between single- and double-camera setups to evaluate whether employing multiple camera perspectives offered any significant advantages in terms of ArUco tracking consistency. Overall, it was determined through the one-tailed paired t-test that a double-camera setup outperformed the single-camera setup by 6.3 ± 0.9% of identified frames (*p *< 0.0001^a^) (Fig. 2*C*). From each individual camera's perspective, an average of 9,177.13 (15.5%) total frames were identified by Camera #1, and 36,706.33 (61.9%) total frames were identified by Camera #2 exclusively. Considering the better-performing camera of the two, we report an average of 36,706.33 (61.9%) total frames identified within a single-camera setup out of 59,310.27 average total frames. In contrast, an average of 40,459.87 (68.3%) total frames was identified from both cameras inclusively. In our setup, the decrease in total frames identified could be contributed, in part, by motion blur. Applying Equation 6 with our experimental parameters – 60 frames per second, a 42 cm field of view, and a 1,920-pixel resolution – along with the blur kernel sizes of 2 pixels for nondeteriorated detection and 6 pixels for complete deterioration (as outlined in Hu et al., 2019), we determined the nondeteriorated velocity threshold to be approximately 3 cm/s, with complete deterioration occurring at ∼8 cm/s. This meant that the ArUco marker could be tracked reliably at a velocity lower than 3 cm/s but with consistency declining to a limit of 8 cm/s. Even though the maximum count of detected frames is less than 75%, it does not impact the overall scoring performance, as our data revealed only three instances of false negatives (0.3%). This is because engagement classifications only necessitate one detected frame during a 6 s active trial, granting a margin of resilience for nondetected frames. Additionally, the performance benchmarks for testing computational resource load under both single- and double-camera setups revealed no significant impact on the computer while utilizing the second camera. Compared to the computer's idle state, the total CPU usage increased by ∼28%, while the total memory usage increased by ∼8%, regardless of the camera configuration (Table 2). While these findings are context-specific to our behavioral chamber configuration, they underscore the potential benefits of gaining multiple vantage points while utilizing a marker-based tracking approach.

### Animal engagement performance

**Figure 3.**
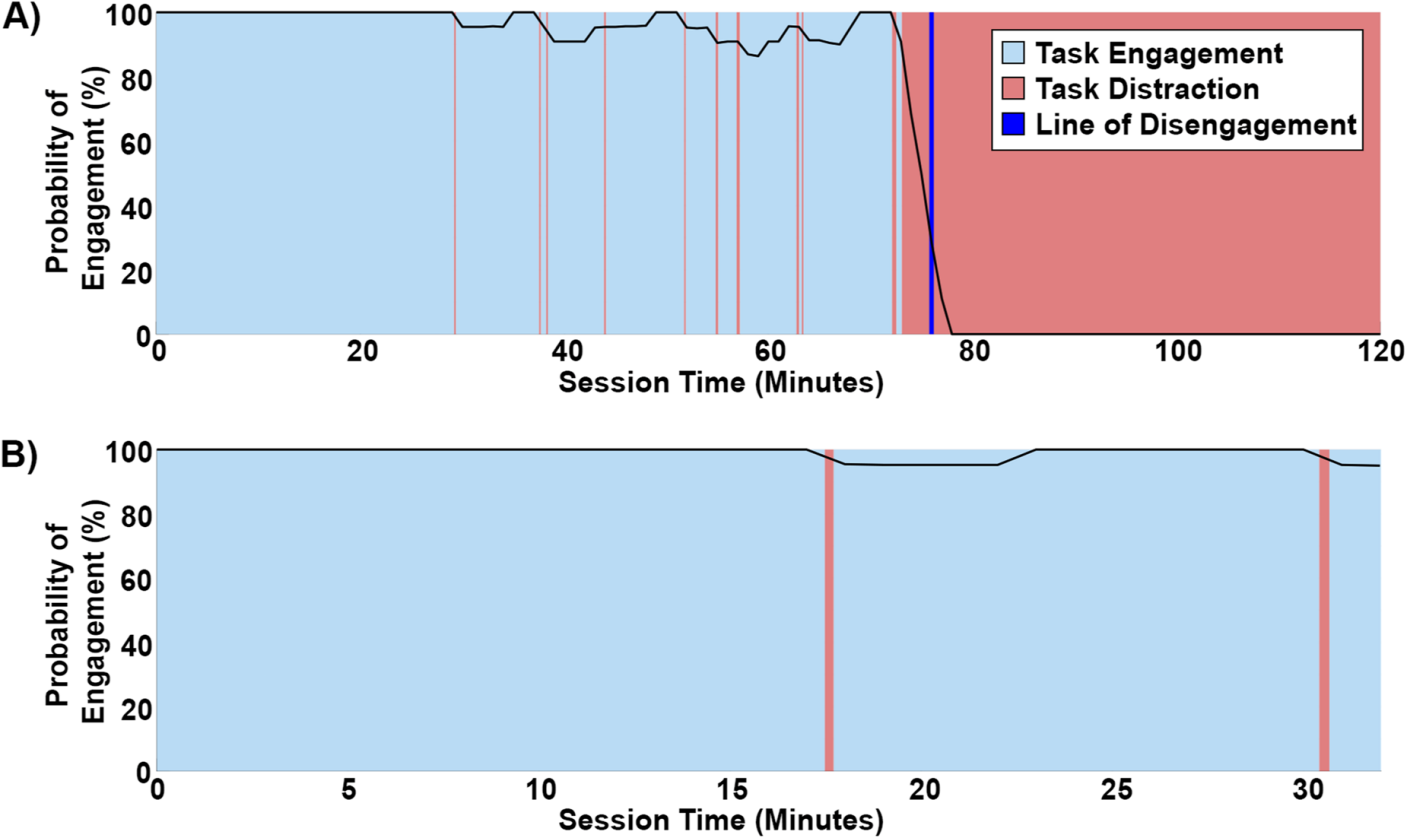
Convolution analysis of task engagement. ***A***, Representative example of engagement dynamics during a 2-h-long nose-poking go/no-go behavioral session. ***B***, Representative example of engagement dynamics during a 32 min nose-poking go/no-go behavioral session.

Our convolution analysis with a 5 min rectangular kernel and a 50% engagement threshold demonstrated the ability to objectively identify transition points between animal engagement and distractions during a go/no-go task. During three of the 2-h-long behavioral sessions (one session per animal), the animals initially maintained attention above the probability of engagement threshold. However, there were noticeable declines at the 75.3 ± 2.3 min mark, indicating a significant shift toward distraction and complete disengagement imposed by our predefined threshold (Fig. 3*A*). In contrast, three of the typical 32 min behavioral sessions displayed constant engagement from the animals throughout their entirety (Fig. 3*B*). These results suggest that our 32 min sessions were set within a reasonable range for achieving robust data through consistent behavioral engagement and could potentially expand to a full hour of behavior without compromising data quality.

**Table:** 

	Data Structure	Type of test	Power
a	Normal Distribution	One-Tailed Paired T-Test	95% CI: 4.517 to 8.151

## Discussion

In this study, we showcased the utility of ArUco markers for real-time assessment and automated scoring of rodent engagement during a nose-poking go/no-go behavioral task. This innovative approach addresses the challenges of quantifying animal behavior with high accuracy and precision, while also providing estimations of the critical transition points between states of engagement and distraction.

While ArUco markers offer an economical and accurate tracking solution, they are subject to certain hardware and setup constraints. For instance, the direct visibility of markers by the camera is imperative for accurate tracking (Garrido-Jurado et al., 2014). Whenever a direct line-of-sight is lost between the camera and marker, occlusions may occur, hindering tracking performance (Garrido-Jurado et al., 2014; Hu et al., 2019; Haalck et al., 2020). In this study, we show that multi-camera setups can help to mitigate occlusions, but in more complex setups or paradigms, two cameras may not be available or enough to ensure consistent tracking. In such cases, we suggest utilizing the program's capacity for multi-marker tracking by modifying the marker mount to accommodate multiple adjacent markers with surfaces oriented at different angles. Although not tested in this study, an example could be a tri-marker approach with adjacent surfaces facing (1) the original upright position, (2) one angled to the left of the animal, and (3) one angled to the right of the animal. This approach offers the advantage of additional viewing angles to address instances of occlusion. However, it comes with the drawback of a bulkier device that animals may be able to scratch or pull, potentially putting strain on the headcap. Furthermore, it is essential to note that the number of USB host controllers a computer possesses imposes bandwidth limitations on multi-camera streaming. In our specific PC configuration, we determined that the maximum number of cameras we could reliably use was three while all the behavioral chamber hardware was connected and running. Although additional USB host controllers can be added to the PC through PCIe USB hubs, this emphasizes the need to balance hardware limitations with tracking consistency when deploying multi-camera setups within a single computer approach. Furthermore, the precise calibration of each camera's position and orientation is critical for accurate chamber-relative tracking; permanent markers on the apparatus can provide a consistent reference, but their placement and calibration demand careful attention (Hu et al., 2019). For instance, we found that Camera #2 was more consistent at identifying the ArUco marker throughout a behavioral session than Camera #1 by 46.4% even though both devices were identical. This mismatch was a result of the two behavioral chamber modules being located on the leftmost half of the chamber's wall; since the animal spent most of its time interacting with the modules, the left-mounted camera (Camera #2) held a better overall viewing perspective. Moreover, the camera specifications themself are important for real-time tracking while attached to an animal. Many studies may wish to opt for economical webcams despite the potential benefits of enhanced resolution and frame rates (Alabbas et al., 2023). Additionally, the durability of the physical ArUco marker is another consideration. Although robust materials like plastic can enhance marker resilience, wear and tear over time is a concern, especially in long-duration experiments where the animals are monitored 24/7. Lighting conditions pose another constraint on tracking. It has been shown that under dimmer or variable lighting conditions sub three lux, tracking performance significantly declines (Hu et al., 2019). This limits our approach to behavioral paradigms that ensure adequate lighting. Although not tested here, some studies have implemented retroreflective materials over the ArUco marker that can be detected by infrared-capable cameras under dark conditions (Ehambram et al., 2019). However, our study operated at a minimum illumination level of ∼27 lux, which ensured reliable tracking results without the need for additional materials. In addition to ensuring adequate lighting conditions, the contrast between the ArUco marker's edges and the background color is crucial. In studies involving Long-Evans or Brown Norway rats, if the fur color is too dark, there might not be sufficient contrast for the ArUco tracking program to identify the marker border. To address these challenges, we recommend printing a thin white border around the ArUco marker pattern. Software limitations further influence the program's tracking performance. Without camera-specific intrinsic calibration, the algorithm defaults to 2D localization. However, simple calibration tools available through the OpenCV library can enable 3D pose tracking, enhancing accuracy in 3D space. Motion blur was another limitation to consider. At various times, we found that our rats could move faster than 8 cm/s, which exceeded the blur kernel ranges for ArUco marker detection. Despite rapid animal movements exceeding these velocities, the global reference frame used in marker tracking allows for swift recovery and accurate trajectory interpolation between tracked positions. For future implementations of our tracking program involving animal movement velocities exceeding 8 cm/s, we suggest considering higher frame rate cameras to potentially enhance tracking reliability. Alternatively, a redesign of the behavioral task/chamber could be implemented to enforce temporarily slower movement speeds or longer trial times, improving the likelihood of marker detection. Finally, marker distance from the camera can affect accuracy. Although not assessed in this study, it has been shown that errors under 2% yaw and 3% translation can occur when the marker is up to 15 times its size away from the camera (Sampathkrishna, 2022). Altogether, researchers should be mindful of these hardware, setup, and software limitations when implementing ArUco marker-based tracking systems in their experiments to ensure optimal performance and accurate behavioral data collection.

ArUco markers offer several advantages for behavioral research; their simplicity and efficiency make them an accessible and scalable solution for real-time tracking, with the potential for tracking multiple animals simultaneously. In contrast to well-established open-source markerless approaches, such as DeepLabCut (Mathis et al., 2018; Nath et al., 2019) and SLEAP (Pereira et al., 2022), ArUco markers offer a robust CPU-driven tracking framework without requiring high-end GPUs for uncompromised performance. This enhanced accessibility extends the utility of ArUco markers to a broader research community. However, we recognize that our ArUco tracking program primarily excels in basic movement and orientation studies, whereas systems like DeepLabCut allow for the complex tracking of multiple body parts capable of providing a more detailed behavioral analysis. When selecting a tracking approach, one should consider the desired data output. In our study, we found that the ArUco tracking program demonstrated a classification accuracy of 98% that was validated against the manual curation of video data. Although specificity should still be improved, this level of accuracy aligns with the performance of the markerless systems, but without the added cost of manually labeling ∼200+ video frames to produce a working model (Mathis et al., 2018; Pereira et al., 2022). However, our comparisons should not end with markerless systems alone. Turning to commercially available high-end marker-based approaches, the Vicon system, a sophisticated motion capture system widely used as the gold-standard for high sub-millimeter precision tracking in various research domains, operates effectively in low-light conditions without compromising performance (Merriaux et al., 2017). This system relies on strategically placed reflective ball markers on the subject, allowing the system to precisely capture and analyze the 3D movement of those markers in real-time. Although effective, Vicon requires costly proprietary hardware and software, limiting its accessibility to many research laboratories. Another commonly used and commercially available contender, Plexon CinePlex, offers multiple tracking modes, including LED, color, and contour tracking for rodents (Jacobson et al., 2014; Xu et al., 2018; Fustiñana et al., 2021). However, this system still possesses some of the common pitfalls of marker-based tracking. The CinePlex Studio v3.7.1 User Guide notes that tracking challenges may arise when dealing with multiple animals/markers of the same color, movement speeds exceeding 200 fps, cameras lacking a direct line-of-sight, and markers positioned either too closely or too far away from the camera. Additionally, it is crucial to recognize that applying a dye to the animal for color tracking poses another potential drawback, particularly in chronic studies where variations in dye placement may occur. Nonetheless, this system also requires costly proprietary hardware and software that limit its accessibility when compared to ArUco.

The successful application of ArUco markers in assessing rodent engagement has several important implications for behavioral neuroscience research. One key implication is the potential for improving the efficiency of behavioral data collection. ArUco marker-based tracking systems can streamline the process of scoring animal behavior by reducing the need for labor-intensive manual video analysis. This efficiency is particularly valuable when conducting large-scale experiments or long-duration behavioral sessions, as it can help researchers manage and analyze extensive datasets more effectively. Furthermore, our convolution analysis of engagement dynamics revealed a substantial decrease in animal engagement around the 75 min mark during 2-h-long sessions. These insights have practical implications for experiment design and protocol optimization. For example, researchers can use ArUco markers to estimate the optimal task duration for their specific behavioral design, ensuring that behavioral sessions are aligned with practical animal engagement levels. This not only improves data quality by focusing on periods of high engagement but can also help maximize the number of behavioral sessions that are able to be run throughout the day.

### Conclusion

In this study, we have validated a robust and highly accurate methodology for assessing animal engagement during a nose-poking go/no-go behavioral task using ArUco markers. This innovative approach leverages the simplicity and efficiency of ArUco markers to provide automated, accessible, and precise estimations of engagement dynamics. Our findings demonstrate the feasibility of utilizing ArUco markers for real-time tracking and estimating critical transition points in animals’ attention during prolonged behavioral sessions. This work not only validates the utility of ArUco markers but also offers a versatile platform for further investigations into animal behavior and engagement dynamics in various experimental settings by allowing automation of behavioral analysis. Future studies may delve into exploring the impact of marker design, tracking algorithms, and behavioral parameters on animal engagement, enhancing our understanding of producing robust behavioral protocols for nose-poking paradigms.

## Synthesis

Reviewing Editor: Sam Golden, The University of Washington Decisions are customarily a result of the Reviewing Editor and the peer reviewers coming together and discussing their recommendations until a consensus is reached. When revisions are invited, a fact-based synthesis statement explaining their decision and outlining what is needed to prepare a revision will be listed below. The following reviewer(s) agreed to reveal their identity: Samantha White, Rongkang Yin.

Dear Dr. Pancrazi,

Your manuscript has been reviewed by two experts. Both experts have provided positive assessments of your manuscript, and pending point-by-point rebuttal and editorial review of a revised manuscript, suggest the manuscript is potentially suitable for publication in eNeuro. Here I will provide a synthesis of the experts main points, and below that their specific review comments.

The manuscript under review introduces an innovative approach for automated behavior scoring in rats performing a go/no-go task, utilizing ArUco markers for real-time tracking of their position and orientation. This method, as highlighted by both reviewers, offers a potential advancement in the field of behavioral neuroscience by addressing challenges like labor intensity and manual bias in traditional behavioral analyses. Reviewer 1 commends the utility of this simple marker-based approach and appreciates the thorough metrics presented, including computational and hardware requirements. They note the system's potential for reliable tracking, especially in cases where rats are tethered to various systems, making facial feature tracking difficult. This is seen as a potential improvement over current commercial and open-source markerless approaches, offering wide accessibility to various labs due to the clarity of rationale and protocol.

Reviewer 2 reinforces the importance of this study, emphasizing the impressive 98% accuracy in categorizing rodents as 'engaged' or 'distracted'. This high level of precision may enhance the reliability of behavioral evaluations. The methodology is praised for its robust design and detail, marking it as a potentially meaningful contribution to the field. Both reviewers collectively recognize the strengths of the manuscript, including its innovative approach and high accuracy, while also acknowledging the need for minor improvements before publication. The synthesis of these reviews suggests that the manuscript has significant potential to advance the understanding and methodology of behavioral neuroscience, particularly in automated tracking and analysis of rodent behavior.

Reviewer 1:

This manuscript presents the utility of a simple marker-based approach to automated behavior scoring in a rat go/no-go task. The authors utilize ArUco markers, small black and white square grids, and pre-existing ArUco detection software to track the real-time position and orientation of rats in an operant chamber. These measurements were used to identify trials where rats were engaged or disengaged in a microstimulation-based go/no-go task and validated against expert hand-scoring of trials. The authors report that the real-time ArUco scoring of engagement on trials showed high accuracy, precision, and sensitivity compared to the expert trackers, with a generally low computational load. Overall, the authors demonstrate the feasibility of using this marker based system for orientation and position tracking in freely-moving rats. I was impressed with the thoroughness of the metrics included in this paper, particularly of those related to the computational and hardware requirements to consider when implementing this system. Below are some strengths of the manuscript, and a list of questions and suggestions to the authors which addresses a few issues with the system, and may increase the usability of the system by other labs.

Strengths:

1) This manuscript elegantly highlights the strengths of a marker-based approach compared to popular alternatives, including commercially available options and open-source markerless approaches. The clarity of the rationale and of the protocol to use this system makes it seem widely accessible to a range of labs.

2) This system seems promising for a simple and reliable tracking of position and orientation of rats performing operant tasks, especially for rats which are tethered to recording systems, microstim systems, infusion cannula or imaging systems which obscure facial features of rats that are otherwise used for orientation tracking, because orientation is measured by a consistent and robust marker attached to the headcap.

Questions/Suggestions:

1) Early in the manuscript (in the methods) I wondered why two cameras were used and whether one camera would be sufficient to track the ArUco marker, especially when considering potential limitations of other laboratories to mount more than one camera and/or stream data from more than one. This is then addressed in the results and discussion section, and it's noted that two cameras outperform the tracking ability of a single camera. However, there was a large difference in the performance of tracking from each individual camera (~15% vs. ~61%). What orientation was the better performing camera in? The authors should include more detailed information about the relative orientation of the two cameras in the main text (and could perhaps include a note about which camera was the better performer in Figure 1B). This will provide insights about the vast differences in performance of the two cameras, and will be informative for future users if they try a similar hardware set-up.

2) I was intrigued by this manuscript initially because the ArUco system seemed like a potential solution to a common problem in freely-moving animals where tethers can obscure the facial features of rats where are commonly used for markerless tracking. The use of an attachment to the headcap seems like an excellent solution, but it was noted in the manuscript (esp. Figure 1C) that the tether was still a problem in some cases and obscured the ArUco marker itself. Is the software capable of identifying the orientation of more than one ArUco marker? If so, do the authors think it would be possible to utilize two ArUco markers (one angled to the left, one angled to the right off the back of the headcap using a similar holding system as described in the paper) for more reliable tracking, with the idea that if one becomes obscured, perhaps the other will not? This might be an interesting alternative to the use of two cameras. The authors should address this feasibility of the use of more than one ArUco tracker in the main text, and if it is possible, share their insights about the advantages and disadvantages of the approach.

3) The authors use Sprague Dawley rats in this study, which I assume make for easy contrast with a black border ArUco marker against their white fur. Is it possible to use a white border ArUco marker, which might contrast better against dark fur of breeds such as Long-Evans and Brown Norway rats? Or would the black border still be sufficient against dark fur? The authors should address this and provide their insights on this in the manuscript text to increase accessibility of the system for other rat species.

4) There was a section which discussed the communication between behavioral control/tracking with MATLAB and the ArUco tracking with Python via socket (lines 198-210). From the description, it was not clear how often MATLAB triggered communication to Python and vice versa. It's stated that this synchronization helped ensure seamless data collection, but what's the exact significance of the synchronization? (comparing trial windows? Correct vs. incorrect trials?) The authors could make the significance more clear, and should be specific about what events (or how often if it was time-based) triggered the communication between programs.

5) The authors noted that frame blurring was also an issue for rats moving faster than 3cm/s and especially 8cm/s. They address it well by explaining the ability of the ArUco tracking software to reliably identify the marker after occlusions and accurate trajectory interpolation, which is good. But for tasks where rats move rapidly (such as a choice task), is it possible to increase reliability of tracking at quicker velocities? I would expect that this would come in the form of increased frame rates (and therefore a slightly better camera). The authors could increase the usability of this system for other tasks by making some recommendations for how to handle this issue in the main text.

6) Finally, the authors demonstrate the utility of this system in a task that occurs on one side of an operant chamber, with two main ports (the nosepoke and the reward port). It would be nice to understand how this system could be expanded to a more 'complex' task design, and by that I mean a multi-port, multi-wall task (i.e. three nosepoke ports on one side with a reward hopper on another wall). Does the program where ROIs are established allow for multiple ROIs? Could this be adjusted to establish ROIs for different task-relevant regions (ie. task engagement vs. reward collection)? The authors should address this possibility in the main text, again, to perhaps increase the usability of this system by other labs.

Reviewer 2:

The presented study introduces a pioneering method using ArUco markers for monitoring rodent behavior in a go/no-go task, effectively addressing key challenges like labor intensity and bias commonly associated with traditional behavioral analyses. The impressive 98% accuracy in categorizing rodents as 'engaged' or 'distracted' is a notable achievement that significantly bolsters the dependability of behavioral evaluations. The methodology is robustly designed and meticulously detailed.

In light of these qualities, the manuscript represents a meaningful and substantial contribution to the domain of behavioral neuroscience. However, there are some minor concerns that need to be resolved before recommendation for publication.

1. ArUco markers stand out for their simplicity and accessibility, demanding fewer computational resources compared to DeepLabCut, which, albeit more intricate and resource-intensive, offers a more thorough analysis. The primary distinction rests in the range of movement analysis. ArUco markers, limited to tracking specific body parts, are apt for general movement and orientation tracking. Conversely, DeepLabCut excels in scrutinizing complex, detailed behaviors across various body parts. The choice between these methodologies hinges on the specific requirements of the research. For basic movement or orientation studies, ArUco markers are an efficient choice. For intricate behavioral analyses, DeepLabCut is preferable, despite its increased complexity and resource requirements. It is important to for the authors to give a more balanced and fair comparison of their ArUco markers method with alternative approaches like DeepLabCut.

2. When evaluating metrics for categories with uneven distribution, such as the "engaged" (95.15%) and "distracted" (4.85%) classifications in the mentioned study, it's crucial to use metrics that can accurately reflect the performance of the classification system, especially in the presence of imbalanced classes. The F1 score, representing the harmonic mean of precision and recall, is more effective than accuracy in imbalanced datasets as it accounts for both false positives and negatives. Additionally, the Area Under the Receiver Operating Characteristic (ROC) Curve (AUC-ROC) is valuable for appraising performance across various classification thresholds, making it particularly beneficial for studies with imbalanced data.
