# Supplementary material for: Real-Time Assessment of Rodent Engagement Using ArUco Markers: A Scalable and Accessible Approach for Scoring Behavior in a Nose-Poking Go/No-Go Task
Source: eNeuro. 2024 Mar 1;11(3):ENEURO.0500-23.2024. doi: 10.1523/ENEURO.0500-23.2024 (PMC11046262; doi:10.1523/ENEURO.0500-23.2024)
Supplement: Repository Files — Extended Data 1. GitHub Repository Code and Additional Files. This file contains all of the code/software, 3D models, and files that will be provided in the GitHub repository for running the experiments and analyzing the data. Download Repository Files, ZIP file. [file eneuro-11-ENEURO.0500-23.2024-s002.zip › Hardware Specifications/Computer Specifications List.pdf]

SI# BGJB01 Precision 5860 Tower Components List.

Component Description	SKU Number
Intel Xeon W5-2445 (26.25 MB cache, 10 cores, 20 threads, 3.1 GHz to 4.6 GHz Turbo, 175 W)	338-CKZM
Windows 11 Pro for Workstations (6 cores plus), English, French, Spanish, Brazilian Portuguese	619-ARSN
No Microsoft Office License Included	658-BCSB
Precision 5860 Tower 1350W Chassis (DAO 2SATA Flexbay L5.5)	321-BHYT
Heatsink for 175W or higher CPU (5860)	412-BBDQ
NVIDIA RTX A4000, 16 GB GDDR6, 4 DP	490-BJJQ
64GB, 2x32GB, DDR5, 4800MHz, RDIMM ECC Memory	370-AHHG
No Out-of-Band Systems Management	631-BBHM
SATA Upper/Lower Flexbay Assembly included with chassis	340-DCMT
Intel Integrated Storage Controller	403-BCVC
No Hard Drive	400-AKZR
No Hard Drive	400-AKZR
No Hard Drive	400-AKZR
No Hard Drive	400-AKZR
No SATA RAID	780-BBCJ
No Optical Drive	429-ABMS
512GB, M.2, PCIe NVMe, SSD, Class	400-BOGY

No Hard Drive	400-AKZR
No M.2 NVME RAID (C1, C2, C6)	780-BCVL
CMS Software not included	632-BBBJ
Virtual Raid on CPU software driver	409-BCXD
Keyboard not included	580-AADS
No Additional Network Card Selected (Integrated NIC included)	555-BBJO
Qualcomm WCN6856 Wireless Card with Bluetooth	555-BHHS

Wireless card PCIe Riser + Antenna	555-BIDG
Firmware and Driver for Qualcomm WCN6856-DBS + Bluetooth module	555-BJNM
System Power Cord (US 125V, 15A)	450-AMEB
ENERGY STAR Qualified	387-BBLW
EPEAT 2018 Registered (Gold)	379-BDZB
Quick Start Guide placemat, 5860 Tower	340-DJVF
SHIP, PWS, LNK, NO, NO, AMF	340-CBUU
Shipping Material (5860, 7865)	340-DCJU
5860T 1350W Regulatory Label (DAO)	389-FDBB
Dell Additional Software	658-BFPP
Precision 5860 Tower XCTO Base	210-BFNP
Dell Precision TPM	340-ACBY
Resource DVD not Included	430-XXYU
Dell Limited Hardware Warranty Plus Service	882-0661
ProSupport Next Business Day Onsite 5 Years	882-0682
ProSupport 7x24 Technical Support 5 Years	882-0690

Thank you choosing Dell ProSupport. For tech support, visit //support.dell.com/ProSupport	989-3449
No External ODD	429-ABGY
Premier Color 6.1	640-BBSS
CFI, Information, MIAS, Post Burn, Factory Install	362-7806
CFI Titan Code for CFI FIDA or Bypass SI	364-1846
CFI Routing SKU	365-0257
Custom Asset Tag	366-0133
Custom Asset Report	366-0135
CFI, Information Latude or Optiplex, Only	371-2950
CFI, Information, CSRouting, Eligible, Factory Install	375-3088
CFI, Information, Label, Medium, B GJB, Factory Install	376-8803
CFI, Information, Label, Large, BG JB, Factory Install	376-8847
