# Supplementary material for: Real-Time Assessment of Rodent Engagement Using ArUco Markers: A Scalable and Accessible Approach for Scoring Behavior in a Nose-Poking Go/No-Go Task
Source: eNeuro. 2024 Mar 1;11(3):ENEURO.0500-23.2024. doi: 10.1523/ENEURO.0500-23.2024 (PMC11046262; doi:10.1523/ENEURO.0500-23.2024)
Supplement: Repository Files — Extended Data 1. GitHub Repository Code and Additional Files. This file contains all of the code/software, 3D models, and files that will be provided in the GitHub repository for running the experiments and analyzing the data. Download Repository Files, ZIP file. [file eneuro-11-ENEURO.0500-23.2024-s002.zip › MATLAB Behavior Program/MATLAB SDK for PlexStim 2.0 - 64 bit/PlexStim Change Log.pdf]

Neurotechnology Research Systems

PlexStim™ Electrical Stimulator
Change Log

www.plexon.com

PlexStim Electrical Stimulator Change Log v2.3.0

(Last Updated 4/23/2015)

This version of the PlexStim™ Electrical Stimulator software is being released in conjunction with new firmware (Firmware PN 14-20-A-07-A). The software in this package will ONLY work with Plexon Stimulators that have the updated firmware.

Plexon Stimulators of hardware revision F (Hardware PN 14-20-A-10-F) are the first stimulators originally manufactured with Revision A firmware (starting in April 2015). Examine the labels on the bottom of the stimulator to determine the firmware and hardware revision BEFORE attempting to use this software. Contact Plexon support (support@plexon.com) for information on upgrading the firmware if required.

New Features:

- The C/C++ SDKs (32-bit and 64-bit) now support four stimulators simultaneously.
- Six new functions were added to the PlexStim Dynamic Link Library (DLL). See the *PlexStim Electrical Stimulator DLL Guide.*

Improvements:

- The behavior of the stimulator in the "Level" triggered digital input mode has changed.
- Significant changes were made for improved reliability of communication between the host computer and stimulator hardware.
- The *PlexStim Electrical Stimulator User Guide* has been updated and now also includes instructions for importing the PlexStim library to LabVIEW.
- The Graphical User Interface (GUI) was updated to Revision 2.3.0.0 where changes are largely transparent to the user.
- In the C/C++ SDKs (32-bit and 64-bit), the PlexStim DLLs have been updated to Revision 2.3.17.0.

  NOTE: User programs written for previous versions of the PlexStim DLL are likely to require modification before they can function with Revision 2.3.17.0 DLL.

  - Verification of input parameters and pattern files were vastly enhanced.
  - The C/C++ example programs (32-bit and 64-bit) have been updated to reflect changes in the PlexStim library and have been updated to Visual Studio 2010.
- The MATLAB® SDKs (32-bit and 64-bit) and example programs were updated to reflect changes in the PlexStim library.
- Drivers were updated from version 2.8 to version 2.12.

PlexStim v2.2.0

New Features:

- The SDK now supports two stimulators.

Improvements:

- The SDK sorts all connected stimulator devices by their serial number(s) where the smaller serial number always show first.

Bug Fixes:

- Fixed bug where it was not possible to run the GUI or C/C++/MATLAB initiation functions twice in a row without first cycling the power on the stimulator.
- Fixed bug where it was not possible to run the GUI, close it, and then run the MATLAB initiation functions (and vice-versa).
- Fixed bug where the GUI did not shut down correctly in Microsoft® Windows®. An entry for "Stim-2.exe" stayed in the Windows task manager each time it ran until you reboot.
- Fixed bug in MATLAB API for PlexStim Stimulator which caused MATLAB to lock up while trying to run the PS_CloseStim(StimN) function.

About Plexon Inc

Plexon is a pioneer and leading innovator of custom, high-performance data acquisition, behavior and analysis solutions specifically designed for scientific research. We collaborate with and supply thousands of customers including the most prestigious neuroscience laboratories around the globe driving new frontiers in areas including basic science, brain-machine interfaces (BMI), neurodegenerative diseases, addictive behaviors and neuroprosthetics. Plexon offers integrated solutions for *in vivo* neurophysiology, optogenetics, and behavioral research – backed by its industry-leading commitment to quality and customer support. For more information, please visit www.plexon.com.

Sales Support

For Sales Support, email info@plexon.com or call +1 (214) 369-4957.

Technical Support

If after reviewing this document, you would still like to access Plexon's Technical Support, we are available via several communication channels. You are invited to reach us through email, on the phone, or even over Skype utilizing instant messaging, voice, and/or video as follows:

EMAIL	PHONE	INSTANT MESSAGING, VOICE OR VIDEO VIA SKYPE
support@plexon.com	8:30 a.m. to 5:00 p.m. Central Time<br>+1 (214) 369-4957	8:30 a.m. to 5:00 p.m. Central Time<br>Skype name: plexonsupport<br>*Skype is a free service. For more information on Skype or to download the application, go to www.skype.com.*

PLEXON®, the five-line symbol, CereStage™, CineCorder™, CineLAB™, CineLyzer™, CinePartner™, CinePlex®, CineTracker™, CineTyper™, DigiAmp™, MiniDigi™, Offline Sorter™, OmniPlex®, PL2™, PlexBright®, PlexDrive™, PlexStim™, Radiant™ and RapidGrid™ are registered and unregistered trademarks of Plexon Inc, Dallas, Texas, USA. ©2015 Plexon Inc. All rights reserved. Other product and company names mentioned are trademarks of their respective owners.

OFSCL0001b
