# Supplementary material for: Real-Time Assessment of Rodent Engagement Using ArUco Markers: A Scalable and Accessible Approach for Scoring Behavior in a Nose-Poking Go/No-Go Task
Source: eNeuro. 2024 Mar 1;11(3):ENEURO.0500-23.2024. doi: 10.1523/ENEURO.0500-23.2024 (PMC11046262; doi:10.1523/ENEURO.0500-23.2024)
Supplement: Repository Files — Extended Data 1. GitHub Repository Code and Additional Files. This file contains all of the code/software, 3D models, and files that will be provided in the GitHub repository for running the experiments and analyzing the data. Download Repository Files, ZIP file. [file eneuro-11-ENEURO.0500-23.2024-s002.zip › MATLAB Behavior Program/MATLAB SDK for PlexStim 2.0 - 64 bit/PlexStim Electrical Stimulator v2.3 MATLAB API Definitions.pdf]

Neurotechnology Research Systems

PlexStim™ Electrical Stimulator MATLAB® API Definitions

www.plexon.com

PlexStim™ Electrical Stimulator MATLAB® API Definitions

Contents

3 **Introduction**

3 **Definitions by Function Type**

3 Information Functions

4 Initialization/Clean-up Functions

5 Loading Channel Functions

6 Pattern Functions

11 Settings Functions

16 Stimulation Functions

19 **Appendix - Full List of Functions**

Documentation History

Date	Version	Notes	Author
May 2015	STMTN0001b	- Updated definitions to version 2.3 and reorganized by function type.	Tanya Mukhina<br>Yolanda Rowe
March 2014	STMTN0001a	- Reviewed and reformatted document.	Tanya Mukhina<br>Yolanda Rowe
August 2012	v1.0	- Initial creation of document.	Tanya Mukhina

Introduction

The following MATLAB® API Definitions support the PlexStim™ Electrical Stimulator operating software version 2.3. This version of the API supports up to four stimulators. API definitons are grouped by function in alphabetical order.

Definitions by Function Type

Information Functions

Name	Description
PS_GetExtendedErrorInfo	Returns an English language description of the specified error code.
PS_GetDescription	Reads back the description (hardware model) of the stimulator.
PS_GetFwVersion	Gets firmware version number.
PS_GetSerialNumber	Gets the serial number of the stimulator.

Definitions

PS_GetExtendedErrorInfo

Syntax

[ErrorString, err] = PS_GetExtendedErrorInfo (ErrorCode)

Description

Returns an English language description of the specified error code.
ErrorCode - the error code for which you want a description
Returns:
ErrorString - English language description of error
0 - OK

PS_GetDescription

Syntax

[DescriptionString, err] = PS_GetDescription (StimN)

Description

Reads back the description (hardware model) of the stimulator.
StimN - stimulator number to query (starts from 1)
Returns:
DescriptionString: The description (hardware model) of the stimulator
err:
0 - OK
1 - device error

PS_GetFwVersion

Syntax

[Version, err] = PS_GetFwVersion (StimN)

Description

Gets firmware version number.
StimN - stimulator number to query (starts from 1)
Returns:
Version: Firmware version number.
err:
-1 - invalid argument(s)
0 - OK

Note : Current SDK was designed to operate with firmware version 6.

PS_GetSerialNumber

Syntax

[NumberString, err] = PS_GetSerialNumber (StimN)

Description

Gets the serial number of the stimulator.

StimN - stimulator number to query (starts from 1)

Returns:

NumberString: The serial number of the stimulator

err:

0 - OK

1 - device error

Initialization/Clean-up Functions

Name	Description
PS_CloseAllStim	Finalizes work with all available stimulators.
PS_CloseStim	Finalizes work with stimulator StimN.
PS_GetNChannels	Returns maximum number of channels (ChN) for the stimulator StimN.
PS_GetNStim	Gets number of available stimulators.
PS_InitAllStim	Initializes all available stimulators.

Definitions

PS_CloseAllStim

Syntax

err = PS_CloseAllStim ( )

Description

Finalizes work with all available stimulators. Any stimulation in progress is aborted.

Returns:

0 - OK

1 - device error

PS_CloseStim

Syntax

err = PS_CloseStim (StimN)

Description

Finalizes work with stimulator StimN. Any stimulation in progress is aborted.

StimN - stimulator number to finalize (starts from 1)

Returns:

-1 - invalid argument (s)

0 - OK

1 - device error

PS_GetNChannels

Syntax

[NCh, err] = PS_GetNChannels (StimN)

Description

Returns maximum number of channels (ChN) for the stimulator StimN and error code (err)

StimN - stimulator number to get number of channels (starts from 1).

Returns:

-1 - invalid argument(s)

0 - OK

PS_GetNStim

Syntax

[N, err] = PS_GetNStim ( )

Description

Gets number of available stimulators. The maximum number of stimulators you can work with is four.

Returns:

0 - OK

N - updated with the number of avilable stimulators

PS_InitAllStim

Syntax

err = PS_InitAllStim ( )

Description

Initializes all available stimulators and places them in stimulation mode (versus Z test mode).

Returns:

0 - OK

1 - error initializing devices

2 - no stimulators found

Loading Channel Functions

Name	Description
PS_LoadAllChannels	Loads parameters of all channels to the stimulator hardware.
PS_LoadChannel	Loads parameters of channel ChN to the stimulator hardware.

Definitions

PS_LoadAllChannels

Syntax

err = PS_LoadAllChannels (StimN)

Description

Loads parameters of all channels to the stimulator hardware.

StimN - stimulator number (starts from 1)

Returns:

-1 - invalid argument(s)

0 - OK

1 - device error

3 - CRC Error

6 - stimulation pattern(s) is(are) not ready (in case of loading an arbitrary pattern)

PS_LoadChannel

Syntax

err = PS_LoadChannel (StimN, ChN)

Description

Loads parameters of channel ChN to the stimulator hardware.

StimN - stimulator number to configure (starts from 1)

ChN - channel number which parameters will be loaded in the stimulator hardware (starts from 1)

Returns:

-1 - invalid argument(s)
0 - OK
1 - device error
3 - CRC Error
6 - stimulation pattern is not ready (in case of loading an arbitrary pattern)

Pattern Functions

Name	Description
PS_GetArbPatternPoints	Gets X and Y coordinates of a graphical representation of the arbitrary waveform pattern loaded into the selected stimulator and channel.
PS_GetArbPatternPointsX	Gets X coordinates of a graphical representation of the arbitrary waveform contained in the loaded pattern file.
PS_GetArbPatternPointsY	Gets Y coordinates of a graphical representation of the arbitrary waveform contained in the loaded pattern file.
PS_GetNPointsArbPattern	Gets the number of points in a graphical representation of the arbitrary waveform.
PS_GetPatternType	Checks configuration of the specified channel.
PS_GetRectParam	Gets parameters of the rectangular pulse for a channel ChN.
PS_GetRectParam2	Gets bi-phasic rectangular pulse parameters (as an array) for the specified channel.
PS_LoadArbPattern	Loads an arbitrary waveform pattern file.
PS_SetPatternType	Configures channel ChN for bi-phasic rectangular pulse or arbitrary waveform pattern operation.
PS_SetRectParam	Sets bi-phasic rectangular pulse parameters (as an array) for the specified channel.
PS_SetRectParam2	Sets parameters of the rectangular pulse for a channel.

Definitions

PS_GetArbPatternPoints

Syntax

[Coords, err] = PS_GetArbPatternPoints (StimN, ChN)

Description

Gets X and Y coordinates of a graphical representation of the arbitrary waveform pattern loaded into the selected stimulator and channel. These coordinates can be used to draw a graph of the pattern.

StimN - stimulator number to query (starts from 1)

ChN - channel number to query (starts from 1)

Returns:

Coords: array of integer coordinates of the points; it accomodates NPoints*2 integer values;
"Coords" contains coordinates as x1 y1 x2 y2 ....

err:

-1 - invalid argument(s)
0 - OK
5 - NPoints is not equal number of point for the pattern in this channel

PS_GetArbPatternPointsX

Syntax

[CoordsX, err] = PS_GetArbPatternPointsX (StimN, ChN)

Description

Gets X coordinates of a graphical representation of the arbitrary waveform contained in the loaded pattern file.

StimN - stimulator number to query (starts from 1)

ChN - channel number to query (starts from 1)

Returns:

CoordsX: array of X coordinates of the points; it accomodates NPoints of integer values;
CoordsX contains coordinates as x1 x2 x3 ....

err:

-1 - invalid argument(s)
0 - OK
5 - NPoints is not equal number of point for the pattern in this channel

PS_GetArbPatternPointsY

Syntax

[CoordsY, err] = PS_GetArbPatternPointsY (StimN, ChN)

Description

Gets Y coordinates of a graphical representation of the arbitrary waveform contained in the loaded pattern file.

StimN - stimulator number to query (starts from 1)

ChN - channel number to query (starts from 1)

Returns:

CoordsY: array of Y coordinates of the points; it accomodates NPoints of integer values;
CoordsY contains coordinates as y1 y2 y3 ....

err:

-1 - invalid argument(s)
0 - OK
5 - NPoints is not equal number of point for the pattern in
this channel

PS_GetNPointsArbPattern

Syntax

[NPoints, err] = PS_GetNPointsArbPattern (StimN, ChN)

Description

Gets the number of points in a graphical representation of the arbitrary waveform.

StimN - stimulator number to query (starts from 1)

ChN - channel number to query (starts from 1)

Returns:

NPoints: the number of points in a graphical representation of the arbitrary waveform pattern loaded into the selected stimulator and channel

err:

-1 - invalid argument(s)

0 - OK

PS_GetPatternType

Syntax

[Type, err] = PS_GetPatternType (StimN, ChN)

Description

Checks if channel is set up to use rectangular pulse parameters or a preloaded arbitrary waveform pattern.

StimN - stimulator number to query (starts from 1)

ChN - channel number to configure (starts from 1)

Returns:

Type: which pattern type is in use: 0 if rectangular pulse, 1 if preloaded arbitrary waveform

err:

-1 - invalid argument(s)

0 - OK

PS_GetRectParam

Syntax

[Param, err] = PS_GetRectParam (StimN, ChN)

Description

Gets parameters of the rectangular pulse for a channel ChN.

StimN - stimulator number to query (starts from 1)

ChN - channel number to query (starts from 1)

Returns:

Param - array 1x5 containing parameters of the rectangular pulse:

Param [0] is first phase amplitude

Param [1] is second phase amplitude

Param [2] is first phase width

Param [3] is second phase width

Param [4] is interphase delay

Returns:

err:

-1 - invalid argument(s)

0 - OK

PS_GetRectParam2

Syntax

[Param, err] = PS_GetRectParam2 (StimN, ChN)

Description

Gets parameters of the rectangular pulse for a channel ChN. Has the same effect as the function PS_SetRectParam2, but returns parameters of the pulse as a structure having five fields.

StimN - stimulator number to query (starts from 1)

ChN - channel number to query (starts from 1)

Returns:

- Param: structure having five fields:
  - Pattern.A1 is first phase amplitude
  - Pattern.A2 is second phase amplitude
  - Pattern.W1 is first phase width
  - Pattern.W2 is second phase width
  - Pattern.Delay is interphase delay
- err:
  - -1 - invalid argument(s)
  - 0 - OK

PS_LoadArbPattern

Syntax

err = PS_LoadArbPattern (StimN, ChN, S)

Description

Loads an arbitrary waveform pattern from a (.pat) file into a selected channel.

StimN - stimulator number to configure (starts from 1)

ChN - channel number to configure (starts from 1)

S - string, contains full path (not more than 512 characters) of the file with arbitrary waveform pattern

Returns:

- -1 - invalid argument(s)
- 0 - pattern loaded from file successfully
- 7 - length of the file name exceeds 512
- 8 - file doesn't exist
- 9 - file is opened by another process
- 10 - number of points in the pattern exceeds maximum allowed size of 1000
- 11 - file contains invalid value(s)
- 12 - file contains too few lines (min is 3)
- 13 - file contains a mismatched amplitude duration pair

PS_SetPatternType

Syntax

err = PS_SetPatternType (StimN, ChN, Type)

Description

Configures channel ChN to use rectangular pulse parameters (pattern = 0) or preloaded arbitrary waveform pattern (pattern = 1).

StimN - stimulator number to configure (starts from 1)

ChN - channel number to configure (starts from 1)

Type - pattern type to use:

- 0 - for rectangular pulse parameter
- 1 - for preloaded arbitrary waveform pattern

Returns:

- -1 - invalid argument(s)
- 0 - OK

PS_SetRectParam

Syntax

err = PS_SetRectParam (StimN, ChN, Param)

Description

Sets parameters of the rectangular pulse for a channel.
StimN - stimulator number to configure (starts from 1)
ChN - channel number to configure (starts from 1)
Param - array 1x5 containing parameters of the rectangular pulse; it should be defined, for example, as pattern = [100, -100, 25, 25, 25] before calling PS_SetRectParam function.

Param [0] is first phase amplitude
Param [1] is second phase amplitude
Param [2] is first phase width
Param [3] is second phase width
Param [4] is interphase delay
The default values are:

- first phase amplitude = 100 mA
- second phase amplitude = -100 mA
- first phase width = 50 µs
- second phase width = 50 µs
- interphase delay = 25 µs

Returns:

-1 - invalid argument(s)
0 - OK

PS_SetRectParam2

Syntax

err = PS_SetRectParam2 (StimN, ChN, Param)

Description

Sets parameters of the rectangular pulse for a channel. Has the same effect as PS_SetRectParam, but uses a structure as the third input argument instead of an array.
StimN - stimulator number to configure (starts from 1)
ChN - channel number to configure (starts from 1)
Param - structure containing parameters of the rectangular pulse; the structure has five fields:

Pattern.A1 is first phase amplitude
Pattern.A2 is second phase amplitude
Pattern.W1 is first phase width
Pattern.W2 is second phase width
Pattern.Delay is interphase delay

The structure should be defined, for example, as

Pattern.A1 = 100
Pattern.A2 = -100
Pattern.W1 = 25
Pattern.W2 = 25
Pattern.Delay = 25

before calling PS_SetRectParam2 function.

The default values are:

- first phase amplitude = 100 mA
- second phase amplitude = -100 mA
- first phase width = 50 µs
- second phase width = 50 µs
- interphase delay = 25 µs

Returns:

-1 - invalid argument(s)
0 - OK

Settings Functions

Name	Description
PS_GetAutoDischarge	Checks automatic discharge setting.
PS_GetDigitalOutputMode	Gets didital output mode setting of stimulator.
PS_GetMonitorChannel	Gets the channel selected for monitoring.
PS_GetPeriod	Gets period (milliseconds) for channel ChN.
PS_GetRate	Gets repetition rate for a channel in Hertz.
PS_GetRepetitions	Gets number of repetitions - the number of times that the bi-phasic pulse or the arbitrary waveform is repeated for channel ChN.
PS_GetStimPatternDuration	Gets duration of the whole stimulation pattern.
PS_GetTriggerMode	Gets trigger mode for a stimulator StimN.
PS_GetVmonScaling	Gets the scale factor for the voltage monitor.
PS_IsWaveformBalanced	Checks if the stimulation waveform is balanced.
PS_SetAutoDischarge	Enables/disables automatic discharge.
PS_SetDigitalOutputMode	Sets the Digital Output mode for stimulator.
PS_SetMonitorChannel	Selects the channel to monitor.
PS_SetPeriod	Sets repetition period for the specified channel (in milliseconds).
PS_SetRate	Sets repetition rate for the specified channel (in Hertz).
PS_SetRepetitions	Sets number of repetitions - the number of times that the bi-phasic pulse or the arbitrary waveform is repeated.
PS_SetTriggerMode	Sets trigger mode for specified stimulator.
PS_SetVmonScaling	Sets the scale factor for the voltage monitor output.

Definitions

PS_GetAutoDischarge

Syntax

[Value, err] = PS_GetAutoDischarge (StimN)

Description

** SEE WARNING BELOW **

Checks automatic discharge setting.

StimN - stimulator number to query (starts from 1)

Returns:

Value: status of auto discharge setting; 1 if auto discharge is enabled, 0 if auto discharge is disabled

err:

-1 - invalid argument(s)

0 - OK

** WARNING **

Disabling automatic discharge is ONLY recommended in very specific circustances when the stimulator is used with the AStAR™ system. READ THE SYSTEM MANUALS.

PS_GetDigitalOutputMode

Syntax

[Mode, err] = PS_GetDigitalOutputMode (StimN)

Description

Checks if the Digital Output is low or high during the inter-pulse Interval. Each stimulator channel has a dedicated digital output that indicates when stimulation is occurring on that channel. The digital output is always high during the pulse or arbitrary waveform output, but the user can control the state of the digital output during the time in between pulses or arbitrary waveforms. Default value is 1 (low).

StimN - stimulator number to query (starts from 1)

Returns:

Mode - can have the following values:

0 - high

1 - low

err:

-1 - invalid argument(s)

0 - OK

PS_GetMonitorChannel

Syntax

[ChN, err] = PS_GetMonitorChannel (StimN)

Description

Gets number of channel set for display on the voltage and current monitor outplaces for stimulator StimN.

StimN - stimulator number to monitor (starts from 1)

Returns:

Number of the monitored channel (ChN)

err:

-1 - invalid argument(s)

0 - OK

PS_GetPeriod

Syntax

[Period, err] = PS_GetPeriod (StimN, ChN)

Description

Gets period (milliseconds) for channel ChN.

StimN - stimulator number to query (starts from 1)

ChN - channel number to query (starts from 1)

Returns:

Period: period value in milliseconds.

err:

-1 - invalid argument(s)

0 - OK

PS_GetRate

Syntax

[Rate, err] = PS_GetRate (StimN, ChN)

Description

Gets repetition rate for a channel in Hertz.

StimN - stimulator number to query (starts from 1)

ChN - channel number to query (starts from 1)

Returns:

Rate - rate in Hertz

err:

-1 - invalid argument(s)

0 - OK

PS_GetRepetitions

Syntax

[NRep, err] = PS_GetRepetitions (StimN, ChN)

Description

Gets number of repetitions - the number of times that the bi-phasic pulse or the arbitrary waveform (loaded from a text file) is repeated for channel ChN.

StimN - stimulator number to query (starts from 1)

ChN - channel number to query (starts from 1)

Returns:

NRep: number of repetitions, can range from 1 to 32767; 0 for an infinite number of repetitions.

err:

-1 - invalid argument(s)

0 - OK

PS_GetStimPatternDuration

Syntax

[Value, err] = PS_GetStimPatternDuration (StimN, ChN)

Description

Gets duration of the whole stimulation pattern.

StimN - stimulator number to query (starts from 1)

ChN - channel number to query (starts from 1)

Returns:

Value: duration of the stimulation pattern in microseconds

err:

-1 - invalid argument(s)

0 - OK

PS_GetTriggerMode

Syntax

[Mode, err] = PS_GetTriggerMode (StimN)

Description

Gets mode for a stimulator StimN to start stimulation.

StimN - stimulator number to query (starts from 1)

Returns:

Mode - can have the following values:

0 - sets stimulator StimN to start stimulation from software

1 - stimulation begins when the digital input for the channel transitions from low (~0V) to high (~5V)

2 - Stimulation begins when the digital input transitions from low (~0V) to high (~5V), but if the digital input is still high when the stimulation protocol completes then the stimulation protocol will begin again

err:

-1 - invalid argument(s)

0 - OK

PS_GetVmonScaling

Syntax

[Scaling, err] = PS_GetVmonScaling (StimN)

Description

Gets the voltage monitor scaling for stimulation mode.

StimN - stimulator number to monitor (starts from 1)

Returns:

Scaling: scaling parameter (0.25 V/V, 2.5 V/V, 25 V/V, or 250 V/V)

err:

-1 - invalid argument(s)

0 - OK

PS_IsWaveformBalanced

Syntax

[Value, err] = PS_IsWaveformBalanced (StimN, ChN)

Description

Checks if the stimulation waveform is balanced. Both rectangular pulses and arbitrary patterns should be charge balanced. It means that the same amount of current is deposited and withdrawn from the electrode. This function analyzes the pulse or pattern to check the net charge is not zero. Gets duration of the whole stimulation pattern.

StimN - stimulator number to query (starts from 1)

ChN - channel number to query (starts from 1)

Returns:

Value: 1 if the waveform is balanced (net charge is zero), 0 otherwise

err:

-1 - invalid argument(s)

0 - OK

PS_SetAutoDischarge

Syntax

err = PS_SetAutoDischarge (StimN, Enable)

Description

** SEE WARNING BELOW **

Enables/disables automatic discharge.

Default = ENABLED.

StimN - stimulator number to configure (starts from 1)

Enabled - 1 to enable the discharge; 0 to disable the discharge

Returns:

-1 - invalid argument(s)

0 - OK

** WARNING **

Disabling automatic discharge is ONLY recommended in very specific circustances when the stimulator is used with the AStAR system. READ THE SYSTEM MANUALS.

PS_SetDigitalOutputMode

Syntax

err = PS_SetDigitalOutputMode (StimN, Mode)

Description

Sets Digital Output mode to low or high during inter-pulse interval. Each stimulator channel has a dedicated digital output that indicates when stimulation is occurring on that channel. The digital output is always high during the pulse or arbitrary waveform output, but the user can control the state of the digital output during the time in between pulses or arbitrary waveforms by using this function. Default value is 1 (low).

StimN - stimulator number to configure (starts from 1)

Mode - can have the following values:

0 - high

1 - low

Returns:

-1 - invalid argument(s)

0 - OK

PS_SetMonitorChannel

Syntax

err = PS_SetMonitorChannel (StimN, ChN)

Description

Selects one channel for display on the voltage and current monitor connectors for the stimulator StimN.

StimN - stimulator number to monitor (starts from 1)

ChN - channel to monitor (starts from 1)

Returns:

-1 - invalid argument(s)
0 - OK
1 - device error

PS_SetPeriod

Syntax

err = PS_SetPeriod (StimN, ChN, Period)

Description

Sets period for a channel in milliseconds. Default value is five ms.

StimN - stimulator number to configure (starts from 1)

ChN - channel number to configure (starts from 1)

Period - period value in milliseconds, valid values are from 0.020 ms <= Period <= 125,000 ms

Returns:

-1 - invalid argument(s)
0 - OK

PS_SetRate

Syntax

err = PS_SetRate (StimN, ChN, Rate)

Description

Sets repetition rate for a channel in Hertz. Default value is 200 Hz.

StimN - stimulator number to configure (starts from 1)

ChN - channel number to configure (starts from 1)

Rate - rate in Hertz; valid values are from 0.008 Hz <= Rate <= 50000 Hz

Returns:

-1 - invalid argument(s)
0 - OK

PS_SetRepetitions

Syntax

err = PS_SetRepetitions (StimN, ChN, NRep)

Description

Sets number of repetitions - the number of times that the bi-phasic pulse or the arbitrary waveform (loaded from a text file) is repeated for channel ChN. Default value is 1.

StimN - stimulator number to configure (starts from 1)

ChN - channel number to configure (starts from 1)

NRep - number of repetitions, can range from 1 to 32767; use 0 for an infinite number of repetitions.

Returns:

-1 - invalid argument(s)
0 - OK

PS_SetTriggerMode

Syntax

err = PS_SetTriggerMode (StimN, Mode)

Description

Sets mode for a stimulator StimN to start stimulation.
StimN - stimulator number to configure (starts from 1)
Mode - can have the following values:

0 - sets stimulator StimN to start stimulation from software
1 - stimulation begins when the digital input for the channel transitions from low (~0V) to high (~5V)
2 - stimulation begins when the digital input transitions from low (~0V) to high (~5V), but if the digital input is still high when the stimulation protocol completes then the stimulation protocol will begin again

Returns:

-1 - invalid argument(s)
0 - OK

PS_SetVmonScaling

Syntax

err = PS_SetVmonScaling (StimN, Scaling)

Description

Sets the voltage monitor scaling for the voltage monitor output in units of V/V.
StimN - stimulator number to monitor (starts from 1)
Scaling - predefined scaling parameter (0.25, 2.5, 25, or 250)
Returns:

-1 - invalid argument(s)
0 - OK
1 - device error

Stimulation Functions

Name	Description
PS_Abort	Ceases all stimulation for specifieed stimulator immediately (emergency).
PS_AbortAll	Ceases all stimulation for all available electrical stimulators immediately.
PS_ChannelStimStarted	Checks if stimulation is started for channel ChN.
PS_StartStimAllChannels	Starts stimulation for all channels for the stimulator StimN.
PS_StartStimChannel	Starts stimulation for channel ChN..
PS_StopStimAllChannels	Stops stimulation for all channels for the stimulator StimN.
PS_StopStimChannel	Stops stimulation for channel ChN.

Definitions

PS_Abort

Syntax

err = PS_Abort (StimN)

Description

Causes stimulation on stimulator StimN to cease immediately even if there is a pulse or arbitrary waveform in progress. This is in contrast to stopping stimulation by calling PS_StopStimChannel or PS_StopStimAllChannels.

StimN - stimulator number to finalize (starts from 1)

Returns:

-1 - invalid argument(s)
0 - OK
1 - device error

PS_AbortAll

Syntax

err = PS_AbortAll ( )

Description

Causes all stimulation to cease immediately even if there is a pulse or arbitrary waveform in progress. This is in contrast to stopping stimulation by calling PS_StopStimChannel or PS_StopStimAllChannels.

Returns:

-1 - invalid argument(s)
0 - OK
1 - device error

PS_ChannelStimStarted

Syntax

[bStarted, err] = PS_ChannelStimStarted (StimN, ChN)

Description

Checks if stimulation is started for channel ChN.

StimN - stimulator number to configure (starts from 1)

ChN - channel number to start stimulation (starts from 1)

Returns:

-1 - invalid argument(s)
0 - OK
bStarted = 1 if stimulation started for channel ChN, 0 otherwise

PS_StartStimAllChannels

Syntax

err = PS_StartStimAllChannels (StimN)

Description

Starts stimulation for all channels for the stimulator StimN.

StimN - stimulator number to configure (starts from 1)

Returns:

-1 - invalid argument(s)
0 - OK
1 - device error
4 - wrong trigger mode (trigger mode is not set to PS_TRIG_SOFT (value is 0))

PS_StartStimChannel

Syntax

err = PS_StartStimChannel (StimN, ChN)

Description

Starts stimulation for channel ChN with previously configured parameters.

StimN - stimulator number to configure (starts from 1)

ChN - channel number to start stimulation (starts from 1)

Returns:

-1 - invalid argument(s)

0 - OK

1 - device error

4 - wrong trigger mode (trigger mode is not set to PS_TRIG_SOFT (value is 0))

PS_StopStimAllChannels

Syntax

err = PS_StopStimAllChannels (StimN)

Description

Stops stimulation for all channels for the stimulator StimN.

StimN - stimulator number to configure (starts from 1)

Returns:

-1 - invalid argument(s)

0 - OK

1 - device error

4 - wrong trigger mode (trigger mode is not set to PS_TRIG_SOFT (value is 0))

PS_StopStimChannel

Syntax

err = PS_StopStimChannel (StimN, ChN)

Description

Stops stimulation for channel ChN.

StimN - stimulator number to configure (starts from 1)

ChN - channel number to start stimulation (starts from 1)

Returns:

-1 - invalid argument(s)

0 - OK

1 - device error

4 - wrong trigger mode (trigger mode is not set to PS_TRIG_SOFT (value is 0))

Appendix - Full List of Functions

Information Functions	
**Name**	**Description**
PS_GetExtendedErrorInfo	Returns an English language description of the specified error code.
PS_GetDescription	Reads back the description (hardware model) of the stimulator.
PS_GetFwVersion	Gets firmware version number.
PS_GetSerialNumber	Gets the serial number of the stimulator.

Initialization/Clean-up Functions	
**Name**	**Description**
PS_CloseAllStim	Finalizes work with all available stimulators.
PS_CloseStim	Finalizes work with stimulator StimN.
PS_GetNChannels	Returns maximum number of channels (ChN) for the stimulator StimN.
PS_GetNStim	Gets number of available stimulators.
PS_InitAllStim	Initializes all available stimulators.

Loading Channel Functions	
**Name**	**Description**
PS_LoadAllChannels	Loads parameters of all channels to the stimulator hardware.
PS_LoadChannel	Loads parameters of channel ChN to the stimulator hardware.

Pattern Functions	
**Name**	**Description**
PS_GetArbPatternPoints	Gets X and Y coordinates of a graphical representation of the arbitrary waveform pattern loaded into the selected stimulator and channel.
PS_GetArbPatternPointsX	Gets X coordinates of a graphical representation of the arbitrary waveform contained in the loaded pattern file.
PS_GetArbPatternPointsY	Gets Y coordinates of a graphical representation of the arbitrary waveform contained in the loaded pattern file.
PS_GetNPointsArbPattern	Gets the number of points in a graphical representation of the arbitrary waveform.
PS_GetPatternType	Checks configuration of the specified channel.
PS_GetRectParam	Gets parameters of the rectangular pulse for a channel ChN.
PS_GetRectParam2	Gets bi-phasic rectangular pulse parameters (as an array) for the specified channel.
PS_LoadArbPattern	Loads an arbitrary waveform pattern file.
PS_SetPatternType	Configures channel ChN for bi-phasic rectangular pulse or arbitrary waveform pattern operation.
PS_SetRectParam	Sets bi-phasic rectangular pulse parameters (as an array) for the specified channel.
PS_SetRectParam2	Sets parameters of the rectangular pulse for a channel.

Settings Functions

Name	Description
PS_GetAutoDischarge	Checks automatic discharge setting.
PS_GetDigitalOutputMode	Gets didital output mode setting of stimulator.
PS_GetMonitorChannel	Gets the channel selected for monitoring.
PS_GetPeriod	Gets period (milliseconds) for channel ChN.
PS_GetRate	Gets repetition rate for a channel in Hertz.
PS_GetRepetitions	Gets number of repetitions - the number of times that the bi-phasic pulse or the arbitrary waveform is repeated for channel ChN.
PS_GetStimPatternDuration	Gets duration of the whole stimulation pattern.
PS_GetTriggerMode	Gets trigger mode for a stimulator StimN.
PS_GetVmonScaling	Gets the scale factor for the voltage monitor.
PS_IsWaveformBalanced	Checks if the stimulation waveform is balanced.
PS_SetAutoDischarge	Enables/disables automatic discharge.
PS_SetDigitalOutputMode	Sets the Digital Output mode for stimulator.
PS_SetMonitorChannel	Selects the channel to monitor.
PS_SetPeriod	Sets repetition period for the specified channel (in milliseconds).
PS_SetRate	Sets repetition rate for the specified channel (in Hertz).
PS_SetRepetitions	Sets number of repetitions - the number of times that the bi-phasic pulse or the arbitrary waveform is repeated.
PS_SetTriggerMode	Sets trigger mode for specified stimulator.
PS_SetVmonScaling	Sets the scale factor for the voltage monitor output.

Stimulation Functions

Name	Description
PS_Abort	Ceases all stimulation for specifieed stimulator immediately (emergency).
PS_AbortAll	Ceases all stimulation for all available electrical stimulators immediately.
PS_ChannelStimStarted	Checks if stimulation is started for channel ChN.
PS_StartStimAllChannels	Starts stimulation for all channels for the stimulator StimN.
PS_StartStimChannel	Starts stimulation for channel ChN.
PS_StopStimAllChannels	Stops stimulation for all channels for the stimulator StimN.
PS_StopStimChannel	Stops stimulation for channel ChN.

About Plexon Inc

Plexon is a pioneer and leading innovator of custom, high-performance data acquisition, behavior and analysis solutions specifically designed for scientific research. We collaborate with and supply thousands of customers including the most prestigious neuroscience laboratories around the globe driving new frontiers in areas including basic science, brain-machine interfaces (BMI), neurodegenerative diseases, addictive behaviors and neuroprosthetics. Plexon offers integrated solutions for *in vivo* neurophysiology, optogenetics, and behavioral research – backed by its industry-leading commitment to quality and customer support. For more information, please visit www.plexon.com.

Sales Support

For Sales Support, email info@plexon.com or call +1 (214) 369-4957.

Technical Support

If after reviewing this document, you would still like to access Plexon's Technical Support, we are available via several communication channels. You are invited to reach us through email, on the phone, or even over Skype utilizing instant messaging, voice, and/or video as follows:

EMAIL	PHONE	INSTANT MESSAGING, VOICE OR VIDEO VIA SKYPE
support@plexon.com	8:30 a.m. to 5:00 p.m. Central Time<br>+1 (214) 369-4957	8:30 a.m. to 5:00 p.m. Central Time<br>Skype name: plexonsupport<br>*Skype is a free service. For more information on Skype or to download the application, go to www.skype.com.*

PLEXON®, the five-line symbol, CereStage™, CineCorder™, CineLAB™, CineLyzer™, CinePartner™, CinePlex®, CineTracker™, CineTyper™, DigiAmp™, MiniDigi™, Offline Sorter™, OmniPlex®, PL2™, PlexBright®, PlexDrive™, PlexStim™, Radiant™ and RapidGrid™ are registered and unregistered trademarks of Plexon Inc, Dallas, Texas, USA. ©2015 Plexon Inc. All rights reserved. Other product and company names mentioned are trademarks of their respective owners.

STMTN0001b
