# Supplementary figures and images for: Real-Time Assessment of Rodent Engagement Using ArUco Markers: A Scalable and Accessible Approach for Scoring Behavior in a Nose-Poking Go/No-Go Task
Source: eNeuro. 2024 Mar 1;11(3):ENEURO.0500-23.2024. doi: 10.1523/ENEURO.0500-23.2024 (PMC11046262; doi:10.1523/ENEURO.0500-23.2024)

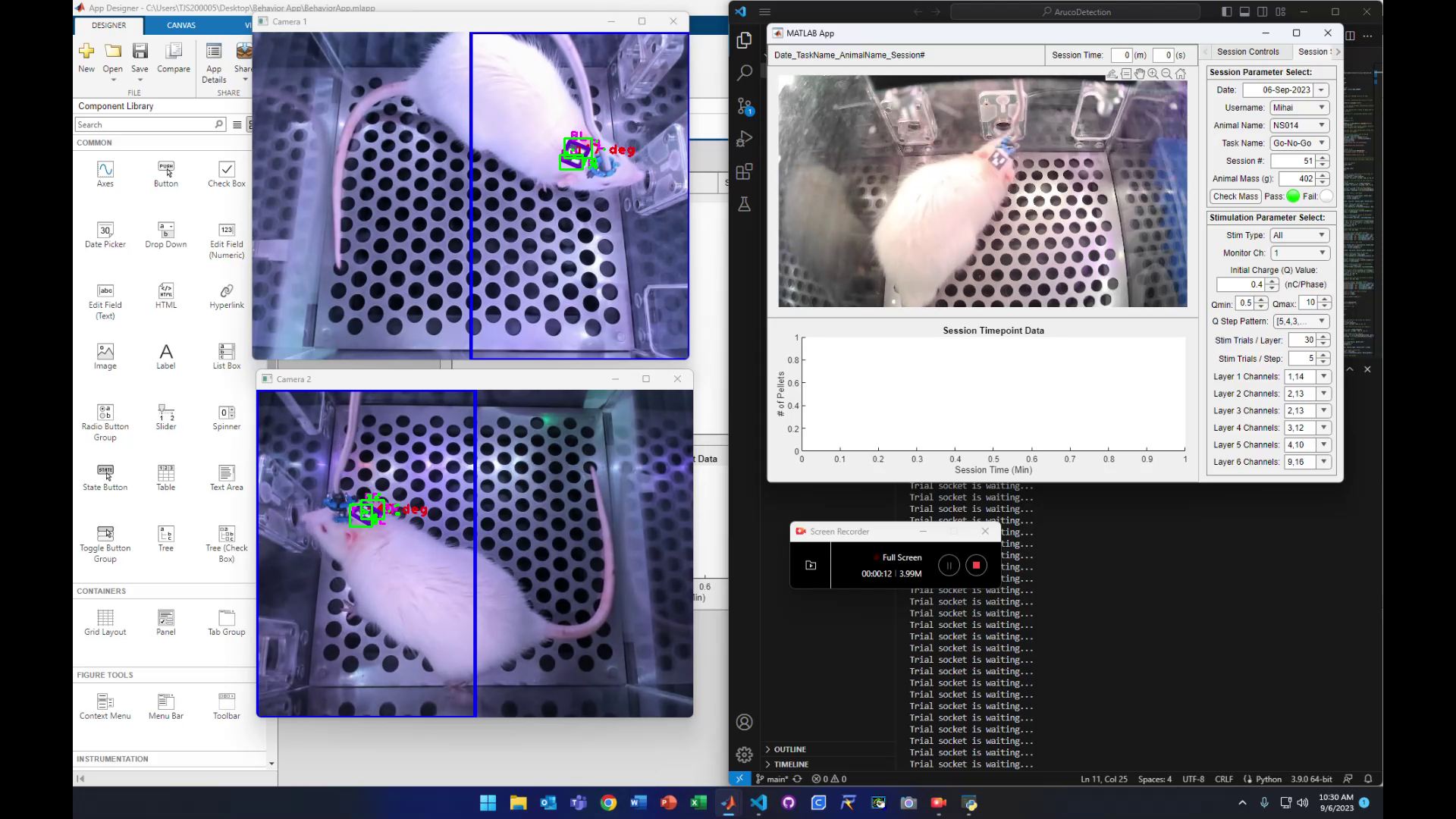

Supplement: Repository Files — Extended Data 1. GitHub Repository Code and Additional Files. This file contains all of the code/software, 3D models, and files that will be provided in the GitHub repository for running the experiments and analyzing the data. Download Repository Files, ZIP file. [file eneuro-11-ENEURO.0500-23.2024-s002.zip › Analysis Code/Old Post-Analysis Frame Reader/Detected Example Frame.png]

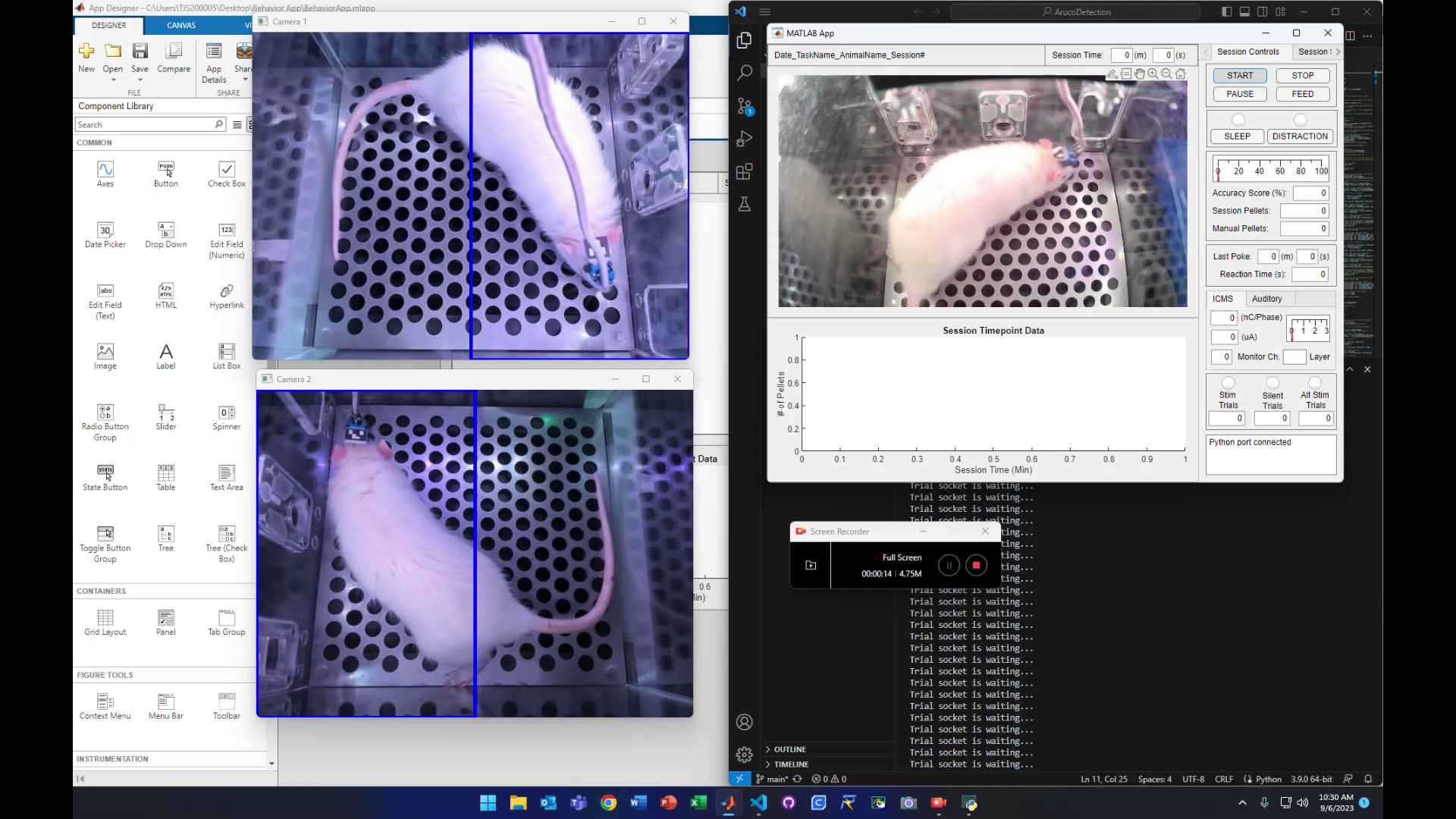

Supplement: Repository Files — Extended Data 1. GitHub Repository Code and Additional Files. This file contains all of the code/software, 3D models, and files that will be provided in the GitHub repository for running the experiments and analyzing the data. Download Repository Files, ZIP file. [file eneuro-11-ENEURO.0500-23.2024-s002.zip › Analysis Code/Old Post-Analysis Frame Reader/Non-Detected Example Frame.png]

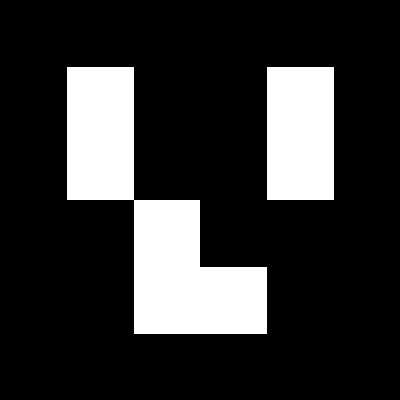

Supplement: Repository Files — Extended Data 1. GitHub Repository Code and Additional Files. This file contains all of the code/software, 3D models, and files that will be provided in the GitHub repository for running the experiments and analyzing the data. Download Repository Files, ZIP file. [file eneuro-11-ENEURO.0500-23.2024-s002.zip › ArUco Tracking Program/marker_image3.png]
